# Transplantation of Human Neural Precursor Cells Reverses Syrinx Growth in a Rat Model of Post-Traumatic Syringomyelia

**DOI:** 10.1007/s13311-020-00987-3

**Published:** 2021-01-19

**Authors:** Ning Xu, Tingting Xu, Raymond Mirasol, Lena Holmberg, Per Henrik Vincent, Xiaofei Li, Anna Falk, Eirikur Benedikz, Emilia Rotstein, Åke Seiger, Elisabet Åkesson, Scott Falci, Erik Sundström

**Affiliations:** 1 Division of Neurogeriatrics, Department of Neurobiology, Care Sciences and Society, Karolinska Institutet, Stockholm, Sweden; 2 Present Address: Center for Reproductive Medicine, and Henan Key Laboratory of Reproduction and Genetics, The First Affiliated Hospital of Zhengzhou University, Zhengzhou, China; 3 Division of Neurogeriatrics, Karolinska Institutet, BioClinicum J10:30, Karolinska University Hospital, S17164 Solna, Sweden; 4 National Institute of Neurological Disorders and Stroke, Stroke Branch, National Institutes of Health, Bethesda, MD USA; 5 Department of Neuroscience, Karolinska Institutet, Stockholm, Sweden; 6 TEK-Innovation, Faculty of Engineering, University of Southern Denmark, DK-5000 Odense, Denmark; 7 Division of Obstetrics and Gynecology, Department of Clinical Science, Intervention and Technology, Karolinska Institutet, S-14186 Stockholm, Sweden; 8 Stockholms Sjukhem Foundation, Box 12230, S-10226 Stockholm, Sweden; 9 Department of Neurosurgery, Craig Hospital, 3425 S. Clarkson St, Englewood, CO 80110 USA; 10 Division of Neurogeriatrics, Karolinska Institutet, BioClinicum J9:20, Karolinska University Hospital, S17164 Solna, Sweden

**Keywords:** Spinal cord injury, Syringomyelia, Animal model, Transplantation, Human neural stem/progenitor cells

## Abstract

Posttraumatic syringomyelia (PTS) is a serious condition of progressive expansion of spinal cord cysts, affecting patients with spinal cord injury years after injury. To evaluate neural cell therapy to prevent cyst expansion and potentially replace lost neurons, we developed a rat model of PTS. We combined contusive trauma with subarachnoid injections of blood, causing tethering of the spinal cord to the surrounding vertebrae, resulting in chronically expanding cysts. The cysts were usually located rostral to the injury, extracanalicular, lined by astrocytes. T2*-weighted magnetic resonance imaging (MRI) showed hyperintense fluid-filled cysts but also hypointense signals from debris and iron-laden macrophages/microglia. Two types of human neural stem/progenitor cells—fetal neural precursor cells (hNPCs) and neuroepithelial-like stem cells (hNESCs) derived from induced pluripotent stem cells—were transplanted to PTS cysts. Cells transplanted into cysts 10 weeks after injury survived at least 10 weeks, migrated into the surrounding parenchyma, but did not differentiate during this period. The cysts were partially obliterated by the cells, and cyst walls often merged with thin layers of cells in between. Cyst volume measurements with MRI showed that the volumes continued to expand in sham-transplanted rats by 102%, while the cyst expansion was effectively prevented by hNPCs and hNESCs transplantation, reducing the cyst volumes by 18.8% and 46.8%, respectively. The volume reductions far exceeded the volume of the added human cells. Thus, in an animal model closely mimicking the clinical situation, we provide proof-of-principle that transplantation of human neural stem/progenitor cells can be used as treatment for PTS.

**Supplementary Information:**

The online version contains supplementary material available at 10.1007/s13311-020-00987-3.

## Introduction

Posttraumatic syringomyelia (PTS), also referred to as progressive posttraumatic cystic myelomalacia (PPCM), is a severe complication of spinal cord injury (SCI) [1], characterized by the formation of cysts which expands rostral and/or caudal to the initial injury during the course of several years. Reliable diagnosis of PTS requires repeated magnetic resonance imaging (MRI) to monitor cyst progression. Symptoms associated with PTS are characterized by progressive motor and sensory deterioration, neuropathic pain, spasticity, and autonomic dysfunction. PTS typically occurs years after injury when neurological symptoms of SCI have stabilized. However, large cysts may develop with no obvious symptoms for many years [2]. An extensive MRI study showed that as many as 30% of persons with SCI are affected by PTS [3]; however, the true prevalence of PTS is unclear, and increasing use of MRI may uncover even higher rates.

Although the pathophysiological process of PTS is not unequivocally established, subarachnoid scarring and tethering of the cord to the surrounding meninges and vertebrae have been recognized as critical components of the development of posttraumatic cysts. In line with this, the surgical treatment includes detethering of the cord by surgical dissection of the subarachnoid scar tissue, with or without shunting of the syrinx to the subarachnoid space or peritoneal cavity [4]. Clinical data show that detethering with duraplasty is more effective than shunting [5], and has become the most commonly used method [6, 7], highlighting the importance of tethering in the pathophysiology of PTS. For some patients, the effect of surgery on cyst expansion is insufficient or transient, in particular patients with extensive tethering [8]. As pointed out by Fehlings and Austin [9], PTS develops over a long time, and longer follow-up times may reveal higher recurrence rates than previously published.

We and others have previously performed clinical trials on patients with PTS, transplanting pieces or tissue suspensions of human embryonic spinal cord [10, 11]. These studies showed that transplantation to PTS cysts is feasible and that transplanted neural tissue survives long term to obliterate the cysts. However, a number of issues regarding logistics and safety limit the use of embryonic tissue for clinical transplantation. In addition, it is not clear if such a transplant prevents further cyst expansion. Transplantation of human neural stem and progenitor cells would obviate many of these concerns. If it can be shown that they prevent or reverse progression of PTS, transplantation of such cells could become a useful adjunct to standard neurosurgical intervention in PTS.

To study treatment of PTS with transplantation of stem/progenitor cells, there are a few rat models of PTS published. In rats, small cysts appear after trauma at the epicenter of the injury. The size of these cavities is typically 2–3 mm^3^ in volume [12, 13]. We also found that occasionally rats with mild to moderate SCI develop larger cysts that progressively expands over time, a rat equivalent to PTS. To develop PTS reliably in rats, several previously published models use injection of foreign material such as kaolin to induce arachnoiditis [14–16]. The presence of foreign material may however directly or indirectly affect transplanted cells making the interpretation of such studies difficult. We therefore replaced kaolin with injection of autologous venous blood to mimic hemorrhage, a clinically well-known cause of arachnoiditis and tethering. After confirming that this procedure leads to appearance of progressively expanding cysts large enough for MRI-guided cell injections in a sufficient proportion of rats, we evaluated if fetal neural precursor cells (hNPCs) and neuroepithelial-like stem cells (hNESCs) derived from induced pluripotent stem cells (iPSCs) could be transplanted to these cysts, their fates, and how successful transplantations affect cyst expansion.

Our hypothesis was that stem/progenitor cells transplanted into the cysts can reverse the expansion of cysts, possibly by adhering to the walls of the cyst, but potentially also through other mechanisms. In addition, due to the inherent potential of neural stem/progenitor cells, we hypothesized that such cells transplanted to PTS cysts can migrate into the host spinal cord, differentiate into appropriate cell types, and as a secondary outcome reconstitute some of the spinal circuitry that has been lost after the injury.

## Material and Methods

### Animals

Adult female Sprague-Dawley rats (Taconic, 180–220 g) were housed in standard cages with controlled temperature and humidity, 12/12 h light-dark schedule, and free access to water and food pellets. Female rats were used to facilitate voiding of urinary bladders after SCI, reducing the risk of urinary tract infection. All animal procedures were in accordance with the Swedish Animal Protection Act and approved by the Regional Ethics Committee on Animal Research, Stockholm, Sweden.

### Animal Surgery and Traumatic Injury

The traumatic SCI was performed as previously reported [17]. #Rats were given atropin (0.05 mg/kg i.p., NM Pharma AB) 30 min before surgery, and anesthetized either with Hypnorm (fentanyl citrate, 0.22 mg/kg plus fluanisome, 6.8 mg/kg, Janssen Pharmaceutical) and dormicum (midazolam, 3.4 mg/kg, Hoffman-La Roche) or isoflurane (4% for induction, 2% for maintenance in a 3:7 mixture of oxygen and air. The body temperature was maintained at 38 °C.

After exposure of the spinal cord at segment Th10–11 (vertebra Th9), a few drops of xylocain (lidocain hydrocloride 20 mg/ml, AstraZeneca) were placed on the spinal cord surface. A mild traumatic injury was induced with an IH spinal cord impactor (Precision Systems and Instrumentation, LLC, 100 kdyn impact, dwell time = 0). Immediately after the injury, 30–100 μl blood from the tongue vein was aspirated with a heparinized 50 μl Hamilton syringe (Sigma-Aldrich). Blood was then slowly injected into the subarachnoid space under vertebra Th8, 2–3 mm rostral to the Th9 laminectomy. The wound was closed in layers, using Lyoplant (B/Brain Aesculap AG) to substitute the dura mater. Rats exposed to the mild traumatic injury without any subarachnoid injections of blood served as controls. To evaluate the therapeutic effect of transplanted hNPCs and hNESCs in the PTS rat model, rats were subjected to the combination of mild trauma and a higher volume of injected blood (50 μl in the first transplantation studies, 100 μl in the intervention study) to increase the chance that cysts would develop. Postoperatively, Temgesic (buprenorphin, 50 μg/kg s.c., Reckitt & Colman) was given twice a day for 4 days. Voiding of urinary bladders was done twice a day until spontaneous evacuation occurred. In the study of functional consequences of PTS, the control group were exposed to the mild traumatic injury without any subarachnoid injections of blood.

### Magnetic Resonance Imaging (MRI)

MRI was performed using a horizontal 9.4 T magnet (Varian, Yarnton, UK) with a 31 cm bore. A 72-mm volume coil was used for transmission, in conjunction with a four-channel phased array surface receive coil (RapidBiomed, Würtsburg, Germany).

The animals were anesthetized using isoflurane and placed in supine position with the spinal cord lesion centered on the surface receive coil. Respiration rate was monitored, and the core body temperature was kept at 37 °C during scanning using a warm air system (SA-instruments, Stony Brook, NY, USA).

The spinal cord was imaged using 2D gradient echo sequence. Respiratory gating was employed, yielding an effective TR of 1.3 s. The remainder of the sequence parameters were TE = 4.35 ms, flip angle = 35°, nex = 4, 35 axial slices of 0.5 mm thickness, field of view 40 mm × 30 mm, and matrix 256 × 192. A spatial saturation band covering the aorta was used to avoid aliasing and motion artifacts from pulsatile flow.

T2*-weighted MRI sequences were used to compare MRI images with sections of post-mortem tissue, identifying T2*-hyper- and hypointense areas in the first part of study. In the cell therapy experiment, MRI cyst volume measurements were done by including T2*-hyperintense, but not hypointense, areas. The cyst volumes were calculated by converting transverse MRI sections to 8-bit format images, and the area of the fluid-filled cyst was measured using MIPAV/Image J software (NIH). To discriminate fluid in the cyst from tissue (graft), grayscale thresholds were set to include pixels representing fluid in the cyst (white), using the muscles surrounding the vertebrae as reference for the grayscale cut-off value. Correct identification of fluid-filled cysts was verified post-mortem by histological examination of the spinal cords. The cyst area of each image was multiplied by the section thickness to calculate the total volume of the cyst.

### Behavioral Assessments

Assessment of motor and sensory functions was performed in two groups of rats, SCI with and without subarchnoidal injection of 30 μl autologous blood, during 20 weeks by a person blinded to the treatment. All rats were pre-trained for the tests used, and all test sessions were video recorded.

Since previously published studies [18] as well as our data showed that the spinal cord cysts developed and expanded from 3 to 6 weeks after injury and onward, we followed motor and sensory functions for 20 weeks.

Motor functions were monitored using (1) the Basso, Beattie, Bresnahan (BBB) locomotor rating scale [19, 20]; (2) the KSAT for swim performance; (3) beam walk assessing the ability to traverse narrow square beams, and (4) grid walk counting misplaced steps.

The BBB scale was used to assess open-field ambulation during 4 min on an elevated 65 × 150 cm surface.

The Karolinska Institutet Swim Assessment Tool (KSAT) [21] was used to assess motor function during swimming. The score, with a maximum of 19 in normal rats, is the sum of the score of six parameters mainly reflecting intensity and frequency of limb and tail movement. All rats were pre-trained to swim to a partially submerged platform at the end of a 150-cm long tank. The tank was filled with tap water (≈ 30 °C) to a depth of 20 cm. The swim parameters were evaluated using video recordings from a lateral position and from below.

In the beam walk test, rats were trained to traverse 1 m beams with an upper rubber surface, widths from 0.7 to 6 cm. Each beam was used for 3 trials, and for each trial, the performance was scored as 0 (unable to traverse the beam), 1 (traversed the beam with > 5 misplaced steps), 2 (traversed with 1–5 misplaced steps), or 3 (no misplaced steps). The average score for each beam was added to give a total score between 0 and 18. Pre-training of rats prior to injury was performed until they repeatedly traversed all beams without any mistakes.

Rats were also evaluated with the grid walk test, described previously [17]. Briefly, rats were trained to cross a 150 × 20 cm grid with 3 × 3 cm holes, and the number of times a hindpaw including the ankle joint slipped through a hole during three trials is counted. The maximum number of mistakes was 47.

Neurogenic pain is a common symptom of PTS, and for this reason, we also measured responses to heat and pressure to determine if the evolving cysts lowered pain thresholds to thermal and mechanical stimuli. To study sensory functions, we determined the thresholds for mechanical and thermal pain. This was not done until 10–20 weeks after injury to avoid the risk that recovery of motor functions by itself would increase responses to painful stimuli. Rats were first habituated to the test situations. Mechanical pain thresholds were analyzed using von Frey filaments (Stoelting) ranging from 0.4 to 447 g [22]. Starting with the thinnest filament, rats were tested by applying increasing pressure on the sole of the hind paws until an avoidance response occurred. This was repeated on each hind paw until the same value was achieved three times. Thresholds to thermal pain were determined using a Plantar test (Hargreaves’) analgesia meter (Ugo Basile). After being habituated to the test cage, the sole of the hindpaws were exposed to the heat source, and the delay until the rat lifted the paw was recorded. The heat was automatically turned off after 15 s to avoid any tissue damage. Each paw was tested 5 times, and the mean delay was calculated. All rats were sensory tested before surgery, and three times between 10 and 20 weeks after surgery.

Since the purpose of the functional testing was to determine the significance of changes occurring at the time of cyst development in the chronic stage, and not the acute-subacute effects of the contusion injury, the statistical analysis was performed using data from 4 weeks after injury and later, i.e., a time when motor functions after the injury had stabilized.

### Human Cell Culture

 hNPCs were cultured as free-floating cell aggregates, so called neurospheres as previously described [23]. Human embryonic spinal cord, 5.5–7 weeks post-conception, was retrieved from elective routine abortions. Dissociated tissue was cultured in DMEM/F12 with glucose (0.6%, Sigma), Hepes (5 mM, Life Technologies), heparin (2 μg/ml, Sigma), N2 supplement (1%, Life Technologies), basic fibroblast growth factor (FGF2, 20 ng/ml, R&D Systems), epidermal growth factor (EGF, 20 ng/ml, R&D Systems), and ciliary neurotrophic factor (CNTF, 10 ng/ml, R&D Systems) at a density of 40,000–50,000 cells/cm^2^ in 20 ml of medium. The NPC were maintained at 37 °C in 5% CO_2_, adding fresh medium twice a week. Neurospheres were passaged every 7 to 10 days by enzymatic dissociation using TrypLE Express (Life Technologies) and mechanical dissociation. Neurospheres at passage 5–10 were used for all experiments.

hNESCs derived from iPSCs were produced by differentiation of the iPSC lines AF22 and C1 as previously described [24, 25]. Briefly, cells dissociated with collagenase were plated in DMEM/F12, NEAA (0.1 mM), β-mercaptoethanol (0.1 mM), L-glutamine (2 mM), and 20% knockout serum replacement. Neural rosette structures were picked, inspected, and transferred to non-adhesive plates with DMEM/F12, L-glutamine (2 mM), glucose (1.6 g/l), penicillin/streptomycin (0.1 mg/ml), and N2 supplement (1:100). Floating aggregates were plated onto cell culture plates coated with poly-L-ornithine and laminin (10 μg/ml, Sigma); grown in the same medium with the addition of FGF2 (10 ng/ml), EGF (10 ng/ml), and B27 (1 μl/ml) (all reagents except laminin from Invitrogen); and passaged every second to third day using trypsin.

hNPC and hNESC cell lines were transduced to express the fluorescent reporter enhanced green fluorescent protein (eGFP). Adherent cells cultured on poly-ornithin/laminin-coated Petri dishes were exposed to 2 μg/ml polybrene and a replication incompetent lentivirus (Allele Biotechnology) carrying the GFP gene driven by the constitutively active EF1α promoter. Estimated MOI was 3–5, and a continuous GFP expression was confirmed using flow cytometry (FACSCalibur, Becton-Dickinson), with a minimum of 90% of cells expressing GFP 1–2 passages before transplantation.

### Cell Transplantation

For the transplantation studies, the size and location of the spinal cord cysts were assessed with MRI 10 weeks after injury. Rats were randomly assigned to transplantation of one of the cell types, or sham transplantation with injection of the same volume of vehicle. All rats were given immunosuppression, ciclosporin (10 mg/kg s.c., Sandimmun, Novartis) once per day, starting 24 h before transplantation/sham transplantation.

Transplantations took place 11–12 weeks after injury, 1–2 weeks after the spinal cysts were localized and measured with MRI. In all transplantation experiments, we avoided hypointense regions in T2*-weighted images. Although these regions often represented potential cyst and injured tissue, they contained tissue debris and phagocytic cells with hemosiderin deposits, which may have negative effects on the transplanted cells.

On the day of transplantation, the rats were again anesthetized, a new skin incision was made, and the vertebral column was exposed at the level of the cyst according to the MRI localization. A glass capillary (0.3 mm end inner diameter) was connected to a 10 μl Hamilton syringe using Teflon tubing, which allowed visual monitoring of the cell suspensions during injection. For transplantation of hNPCs, the capillary was loaded with a suspension of 25–30 neurospheres, diameters 180–400 μm in 7–10 μl of cell culture medium without any growth factors or mitogens. The number of cells in each neurosphere was calculated based on the diameter of the neurospheres, as previously described [17], and a vial with neurospheres containing ≈ 300,000 hNPCs prepared for each rat to be transplanted. hNESCs were transplanted as cell suspensions of 300,000 cells in 3 μl medium. The cyst was punctured, the glass capillary inserted into the cyst, and the cells injected during 15–30 s. Sham-treated rats were injected with 5 μl of the cell culture medium. The glass capillary was carefully removed to minimize leakage of the transplanted cells from the cyst. The injection site was covered with Lyoplant, and the wound closed in layers. At 20 weeks, the spinal cord cysts were again measured with MRI to calculate the volume changes.

### Histological Staining and Immunohistochemistry

At the end of the in vivo experiments, all rats were sacrificed by a lethal injection of pentobarbital (Mebumal vet., Pherrovet AB), transcardially perfused with Ca^2+^-free Tyrode’s solution followed by phosphate-buffered 4% paraformaldehyde (PFA, Merck Millipore). The tissue was post-fixed for 2 h, rinsed, and cryo-preserved in two steps using 10% and 30% sucrose at 4 °C, each for 24 h. The spinal cords including the entire cyst and some spinal cord tissue rostral and caudal to the cyst were sectioned at 10 μm in a cryostat and mounted on glass slides (Superfrost®Plus, Merzel-Gläser).

For bright-field microscopy, tissue sections were stained with hematoxylin and eosin (HE). To detect the presence of Fe^3+^, a modified Prussian blue staining was used [26]. For immunohistochemistry, the following primary antibodies were used: rabbit anti-nestin (1:250, Merck Millipore), rabbit anti-sox2 (1:200, Millipore), rabbit anti-β-tubulin type III (1:1200, Nordic Biosite), rabbit anti-microtubule-associated protein 2 (MAP2, 1:50, Merck Millipore), rabbit anti-GFAP (1:500, Dako Cytomation), human-specific rabbit anti-heat shock protein 27 (hsp-27, 1:1500, Medical & Biological Laboratories), and mouse anti-human nuclear protein (hnp, 1:150, Merck Millipore). Primary antibodies were diluted in 0.01 M phosphate buffer with 0.3% Triton X-100 (TPBS). The secondary antibodies were conjugated to Cy3 (Jackson ImmunoResearch Laboratories Lab. Inc) or Alexa Fluor 488 (Life Technologies). For staining with anti-hnp, epitope retrieval was done by incubating sections in 10 mM sodium citrate buffer at 95 °C for 20 min, then transferred to hot distilled water, and left to cool for 30 min. All sections were pre-incubated in TPBS with 4% bovine serum albumin and 5% normal goat serum (Sigma) for 30 min, incubated with primary antibodies at 4 °C overnight, and rinsed before a 1 h incubation with secondary antibodies at room temperature. All sections were counterstained with nuclear marker Hoechst 33342 (30 μg/ml, Life Technologies).

### Measurement of Graft Cell Number, Cell, and Cyst Volumes

The immunolabeled tissue sections were captured using the Openlab software for MacOS (Improvision) and a CCD camera (Hamamatsu ORCA-ER) connected to the fluorescence microscope (Nikon). Quantification was performed by a person blinded to the identity of the tissue sections.

To estimate the total volume of grafted cells, including cells that migrated into the host tissue, the number of surviving cells was first estimated. Every 10th section was stained with anti-hnp, and the number of labeled cell nuclei was counted in each section. The total number of grafted human cells in each spinal cord was calculated using the Abercrombie equation.

The average volume of grafted cells was measured at × 60 magnification by sampling images of all optical sections through four representative transplanted GFP-expressing hNPCs, using an inverted laser scanning microscope (LSM 510 META, Zeiss, Germany). The cross section areas of adjacent serial sections of each cell were measured using ImageJ, and the added cell areas for each cell are multiplied by the thickness of the optical sections to obtain average cell volume. Using the estimated total number of grafted cells and the calculated average cell volume, an estimate of the total volume of grafted cells was achieved.

In the study on functional consequences of PTS cyst, volumes were measured in serial sections of the spinal cord in the rats used for functional analyses. Since large cysts often collapse during the tissue processing, the perimeter of the cysts was analyzed using Image J software. Since the cysts are close to circular in MRI sections, the measured perimeters were used to calculate the cross section area of a circular cyst with this perimeter, and the cyst volume estimated from the distance between sections (250 μm). Due to the shrinkage of tissue during fixation, the volumes were normalized to the median of the control group (Trauma only).

### Statistics

Data are presented as mean ± standard deviation (SD). Statistical analysis was performed using PASW 18 Statistics software (SPSS). Results of the functional assessment and measurements of cyst volumes were analyzed with ANOVA for repeated measures and post-hoc Bonferroni test. Cyst volumes in post-mortem tissue after functional testing were analyzed with Mann-Whitney *U* test.

### Ethical Permits

Donation of embryonic tissue was preceded by written consent from the pregnant women. All procedures regarding the use of human embryonic cells were approved by the Regional Ethical Committee, Stockholm in accordance with the World Medical Association Declaration of Helsinki 2000, and by the National Board of Health and Welfare. Skin biopsies from healthy volunteers, acquired with permission from the Regional Ethical Committee, Stockholm, were used to produce IPS cells.

No tissue aquired from prisoners was used.

## Results

### Microscopic Evaluation

**Fig. 1:**
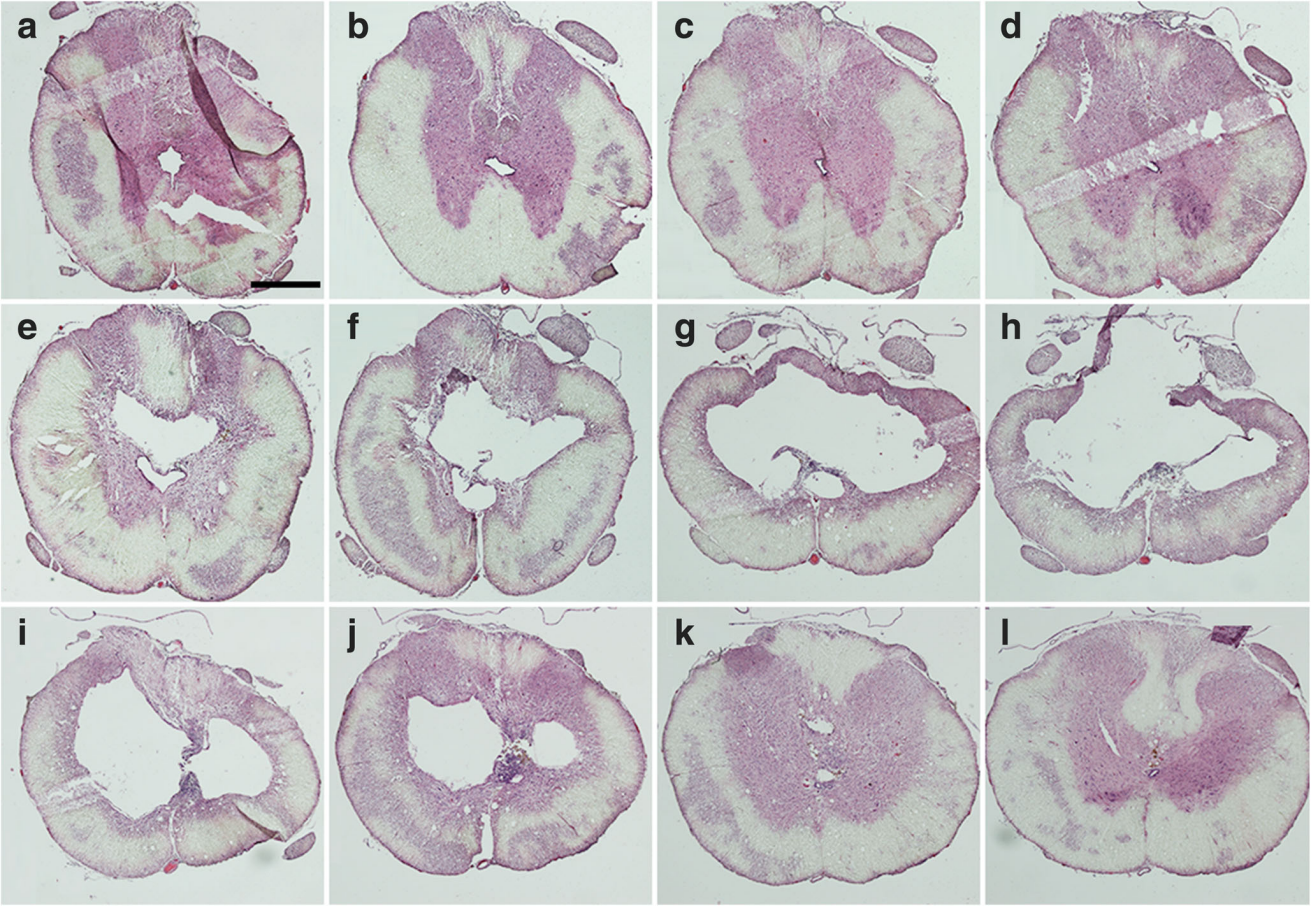
HE-stained cross sections showing a 7 mm long, branched cyst in the thoracic spinal cord, 20 weeks after traumatic SCI combined with subarachnoid injection of 30 μl of venous blood rostral to the contusion (**A**–**L**). Over 2–3 segments of the spinal cord almost all of the gray matter is replaced by the cyst (**F**–**J**). The central canal is typically located ventral to the cyst (**E**–**F**, **J**). The scale bar represents 0.5 mm. The rostro-caudal distance between adjacent images is 1 mm

We performed a series of pilot experiments to evaluate our hypothesis that spinal cord cysts in rats could be produced without exogenous substances. Through a combination of trauma and subarachnoid injection of autologous blood, we mimicked in a standardized way the neural trauma and hemorrhage that occur in patients who go on to develop syringomyelia. In order to minimize the functional deficits caused by the trauma itself and increase the possibility to detect motor symptoms related to the expanding cysts, we used a mild contusion trauma. Mild trauma alone results in moderate degenerative changes at and around the epicenter, myelomalacia with microcysts and reactive astrocytosis, which mainly developed during the first 8 weeks (Suppl. Fig. 1), similar to previous studies [27]. Based on our observations that cysts smaller than 2 mm^3^ spontaneously disappeared (see below), we used this as a criterion of cysts. In 1/5 rats did we find formation of a small cyst (2.5 mm^3^). However, mild trauma immediately followed by injection of 30–50 μl of blood gives rise to macroscopic spinal cord cysts in 50–60% of injured rats sacrificed 12 weeks later (Fig. 1).

**Fig. 2:**
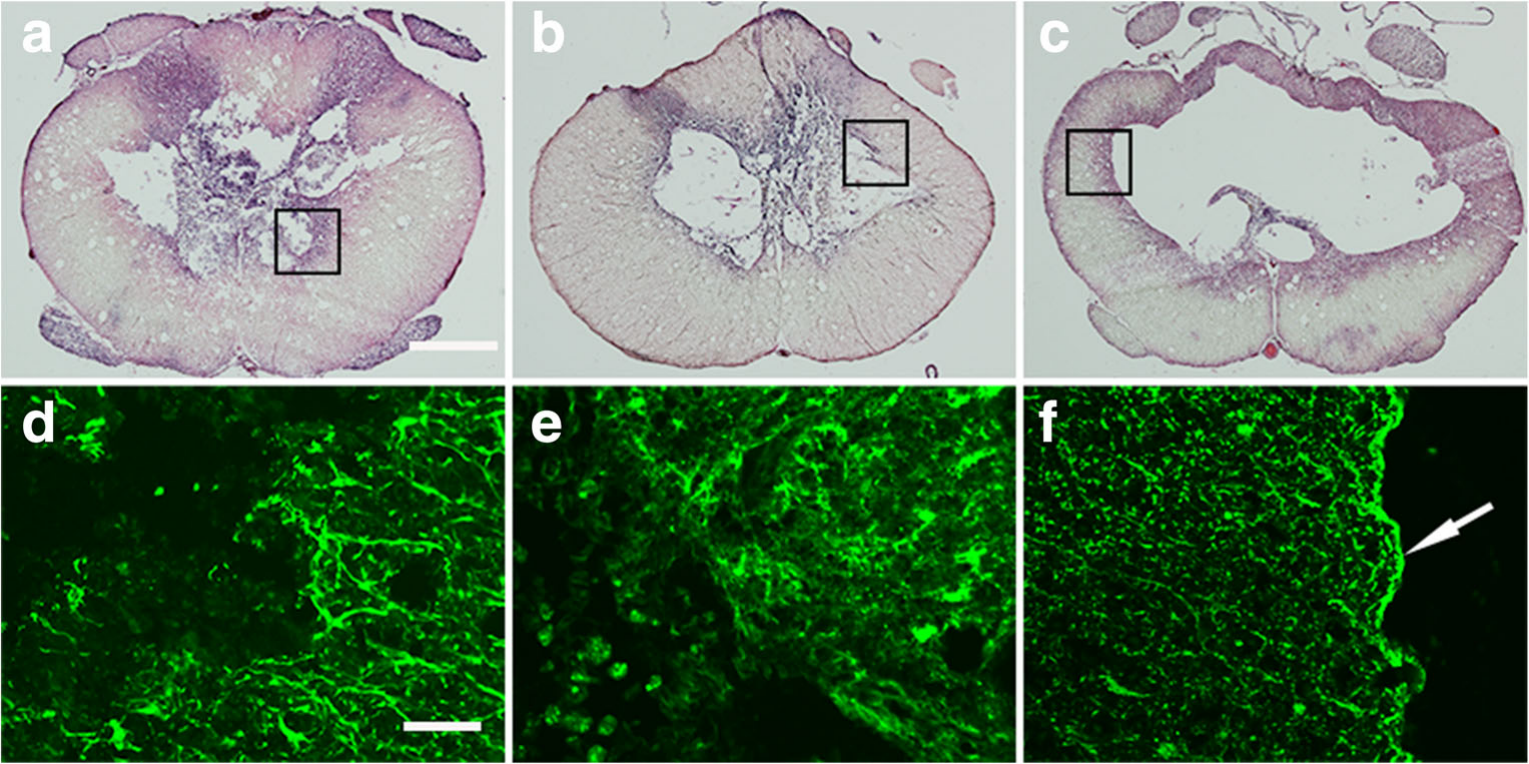
Tissue sections of spinal cords from rats sacrificed 2 weeks (**A**, **D**), 8 weeks (**B**, **E**) and 20 weeks (**C**, **F**) after injury. Sections were HE-stained (**A**–**C**) or immunostained for GFAP (**D**–**F**) to identify reactive astrocytes. The immunostained sections represent the boxed area in the HE-stained sections above them. Scale bars correspond to 0.5 mm (**A**–**C**) and 25 μm (**D**–**F**)

To study the dynamics of cyst formation, we analyzed the histology of the spinal cord and the development of cysts in rats randomly sacrificed 2, 8, and 20 weeks after trauma combined with subarachnoid injection of blood. At 2 weeks after injury, the regions close to the impact mainly contained degenerated tissue. GFAP staining showed reactive astrocytes bordering the lesion. At this stage, it was not possible to decide if there was ongoing development of cysts or just degeneration with microcysts. At 8 weeks, we found cysts in and around the injury site, with debris within the cysts, and reactive astrocytes in the parenchyma surrounding the cysts. Much of the debris is gone at 20 weeks, and the gray matter and degenerative tissue that is typically found at the site of contusion are replaced by cysts, with a surrounding rim of pathological gray matter (Fig. 2). The central canal is usually found intact but widened ventral to the cyst (Fig. 1E–G) except when the area of the central canal was destroyed by the injury and the cyst (Fig. 1H–I). The wider parts of chronic cysts (i.e., with a diameter ≥ 1 mm) were always empty on microscopy examination (presumably fluid-filled before sectioning), but as they narrowed at the rostral and caudal ends, tissue debris could sometimes be found in the cyst. GFAP reactive astrocytes at the injury site are not hypertrophic but formed a clear glia layer lining the cyst cavities (Fig. 2). We found that the mean cyst volume in tissue sections of rats exposed to mild trauma combined with subarachnoid injection of blood was 7.2 times larger than the trauma alone-group (Suppl Fig. 2).

### MRI Imaging

**Fig. 3:**
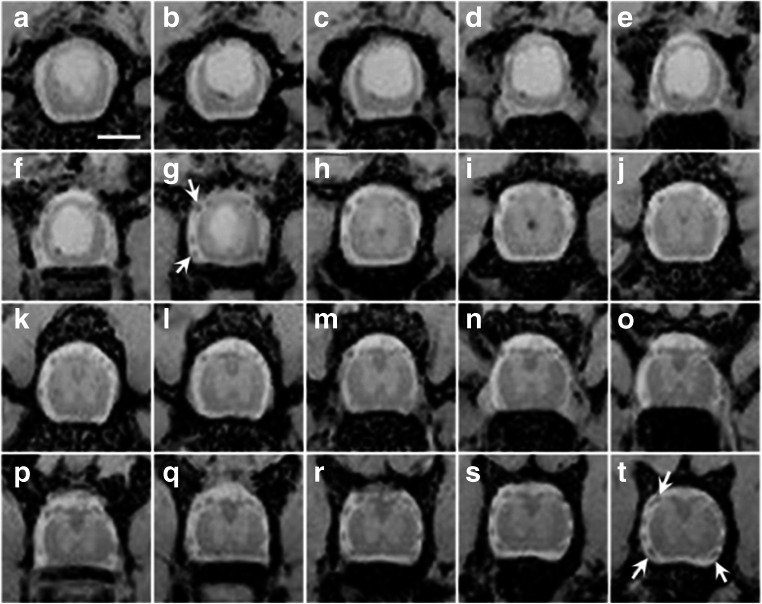
Axial projections of T2-weighted MRI of a rat, 18 weeks after trauma and 30 μl subarachnoid injection of blood, showing the lower 3.5 mm of a large cyst in the thoracic spinal cord, and the round-ovoid structures of tissue in the subarachnoid space at and below the level of the cyst, most obvious in images G–I and P–T (arrows in G and T). These structures probably represent tissue filaments tethering the spinal cord. Each image shows 0.5 mm thick sections of the spinal cord. Scale bar = 1 mm

To determine that the cysts fulfilled the criteria for PTS of continuous long-term expansion, in vivo imaging of the cysts was done in most experiments. A group of 10 rats subject to mild trauma and injections of 30 μl blood were used to investigate development of rat PTS over time. Eighteen weeks after injury, all 9 surviving rats (one sacrificed due to acute complications) had cysts that could be identified with MRI (Fig. 3). Most rats had wide, often branched central T2*-hyperintense cysts. The mean length of T2*-hyperintense cysts was 8.2 mm, with some cysts as long as 13 mm, and the mean volume of cysts in this group was 5.3 mm^3^. In contrast, rats exposed only to the mild trauma typically had minute T2*-hyperintense areas corresponding to small cysts, 1–2 mm long, or no hyperintense signal at all. As expected, the wide part of the cysts usually occurred rostral to the site of contusion. The narrow T2*-hyperintense cysts often extended several millimeters caudal and/or rostral of the main cavity. Six out of nine rats (≈ 65%) had wide (≥ 1 mm diameter) T2*-hyperintense cystic formations that extended over more than one spinal segment. In two rats, the wide part of the cysts did not include more than one segment, and in one rat, there was only a long and very narrow cyst. In addition, T2*-hypointense tissue was seen immediately adjacent to the T2*-hyperintense cysts in axial plane projections and also in the central cord extending up to 10 mm rostral and caudal to the hyperintense parts (Suppl. Fig. 3). It was not possible to determine if the hypointense area represented the central canal or the surrounding tissue.

**Fig. 4:**
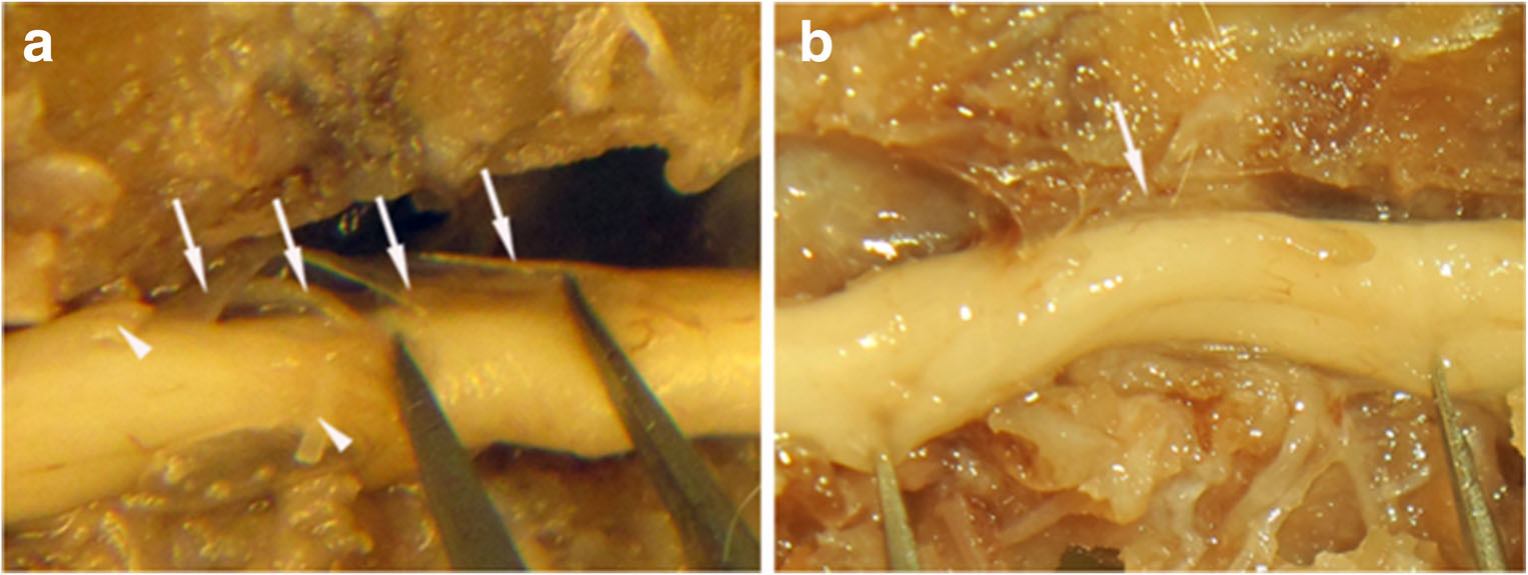
Post-mortem images of the thoracic vertebral column and the spinal cord in two different rats, dissected after intracardial fixation. The ventral vertebrae were removed to expose the spinal cord. The cord is lifted and rotated to expose the dorsal parts where tethering was abundant. In **A**, several bands of scar tissue ran in different directions between the spinal cord and the dura mater (arrows), easily distinguished from the dorsal roots (arrow heads), here cut to avoid confusion. In **B**, a thick sheet of arachnoidal scar tissue (arrow) tethers the spinal cord to the dura mater

Closer examination of the subarachnoid space in the axial MRI images reveals round or ovoid structures occurring over a distance of several millimeters in most rats (Fig. 3), suggesting the presence of strands of connective tissue tethering the spinal cord, a key feature of patients with PTS. At the post-mortem dissection, we found extensive tethering between the spinal cord and the surrounding vertebral column mediated by multiple threads of connective tissue and by more typical scar tissue (Fig. 4).

### Correlation of MRI and Microscopy

**Fig. 5:**
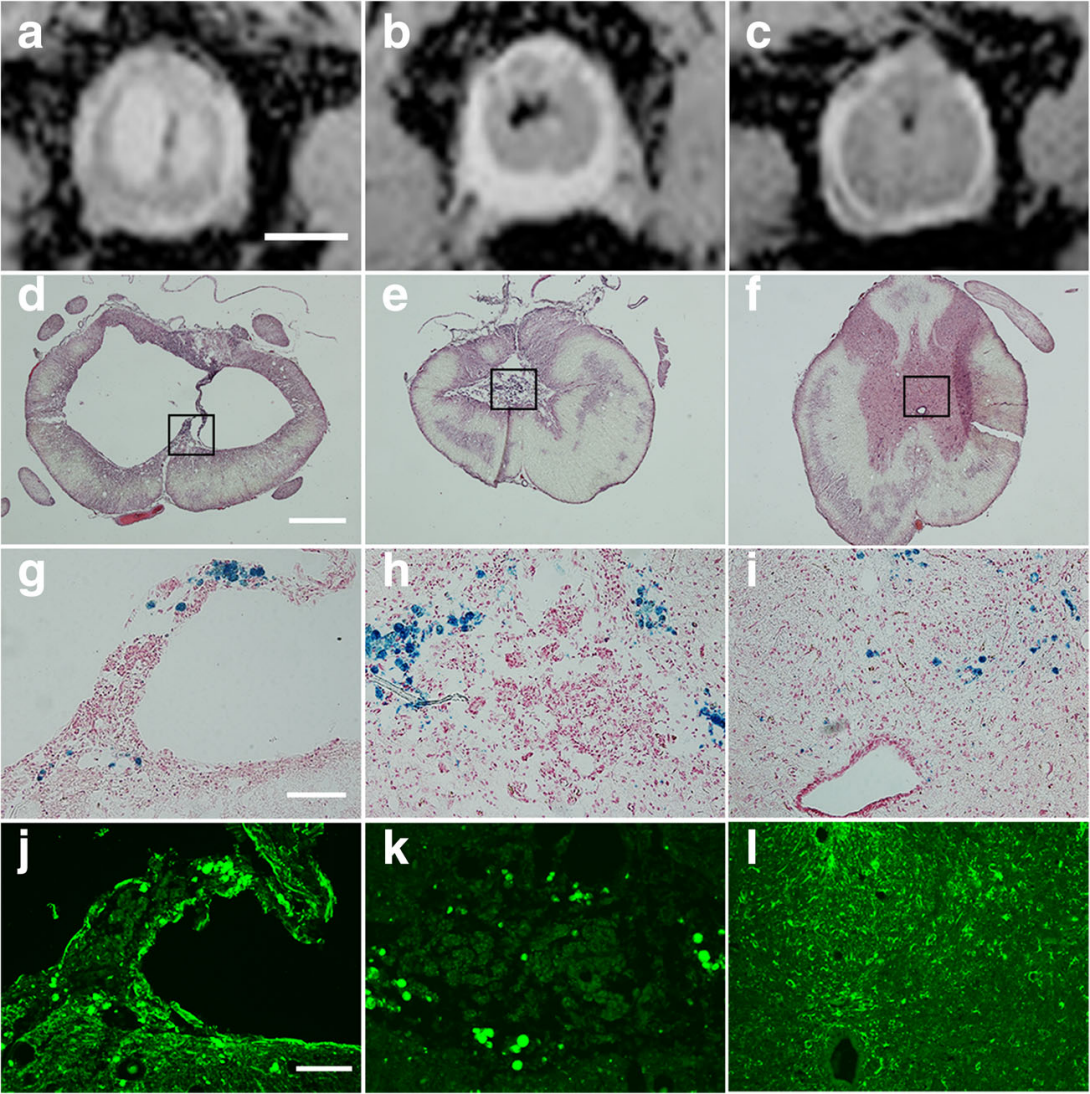
Axial T2-weighted projections of spinal cords illustrating hyper- and hypointense regions in T2-weighted MRI (**A**–**C**), and the corresponding HE-stained tissue sections (**D**–**F**). The boxed areas are shown at higher magnifications applying iron staining (**G**–**I**) and immunofluorescence for GFAP (**J**–**L**). Scale bars correspond to 2 mm (**A**–**C**), 500 μm (**D**–**F**), 50 μm (**G**–**I**), and 100 μm (**J**–**L**)

To further investigate the histology underlying the MRI signal changes, we aligned the tissue sections with axial MRI projections. Hyperintense regions on MRI corresponded as expected to empty cysts in histological tissue sections (Fig. 5A, D). Hypointense regions corresponded to cysts containing debris or to tissue close to the cysts but were also seen in tissue that in HE staining appeared normal. Applying iron histochemistry, the blue reaction product appeared as inclusion bodies in large non-neural cells, presumably macrophages/microglia, and were mainly found in the tissue immediately adjacent to cysts (Fig. 5G), in tissue debris in cysts (Fig. 5H), and in tissue surrounding the central canal, the same regions that appeared hypointense in MRI.

The long, narrow, hypointense structures in some sagittal MRI projections, adjacent to the central canal and usually rostral to the main cyst (Suppl. Fig. 3), appeared as round structures in the central-dorsal gray matter in axial MRI projections. We found no cysts corresponding to these structures in HE-stained tissue sections (Fig. 5F), but iron staining shows iron deposits in the parenchyma at these locations (Fig. 5I). Increased density of GFAP immunostaining was evident in these regions, indicating reactive gliosis and tissue damage (Fig. 5L), despite the undamaged appearance on HE staining. The distribution of iron varied from scattered to aggregated spots but was always found in areas of the parenchyma corresponding to T2*-hypointensity.

As MRI cyst volume measurements were done by including T2*-hyperintense, but not hypointense, areas, the cyst borders at 18 weeks likely underestimated the area of actual tissue damage.

### Functional Effects of Cyst Development

**Fig. 6:**
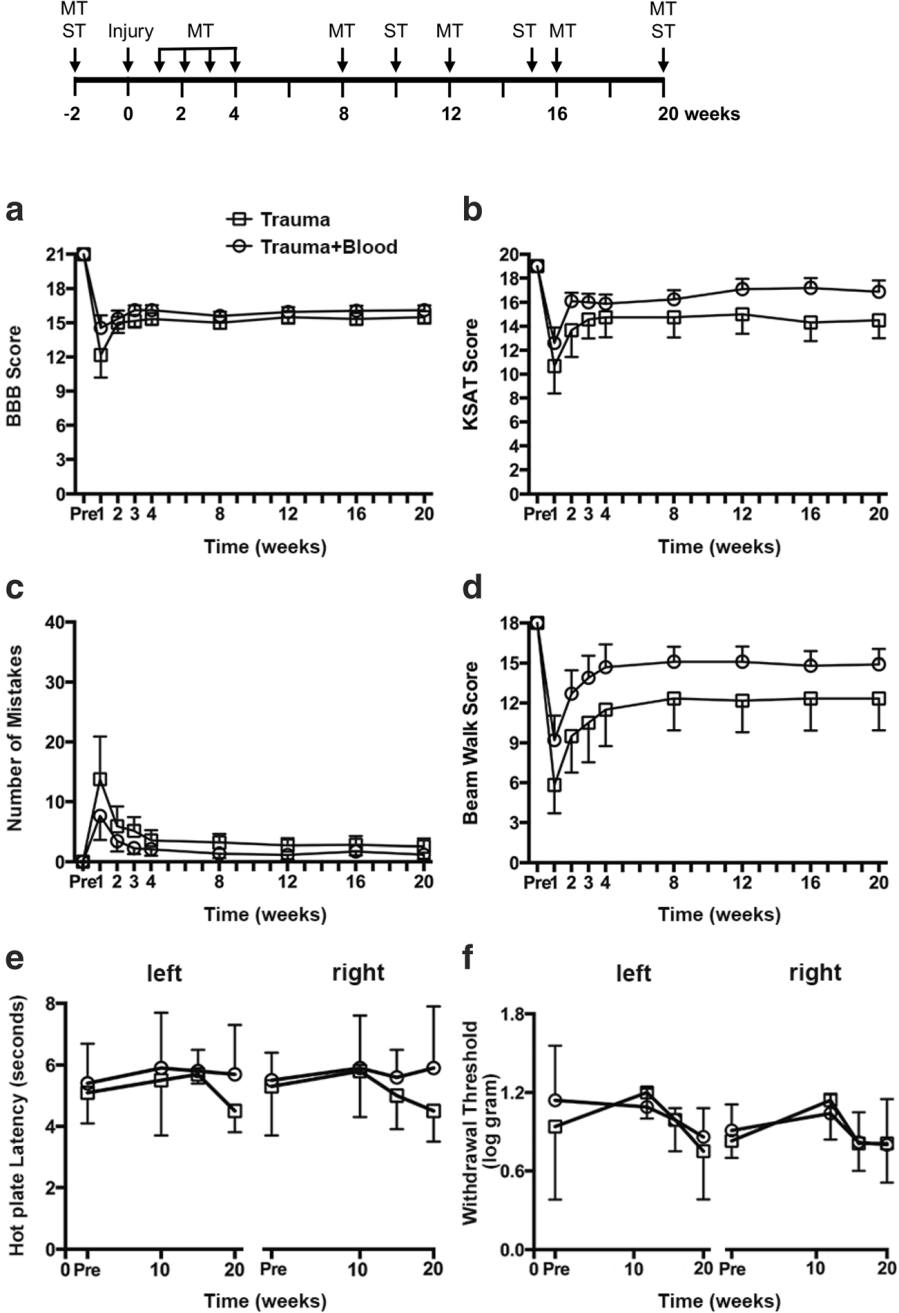
Graphs representing motor and sensory functions before and during 20 weeks after injury in rats exposed either to mild trauma + subarachnoid injection of 30 μl of blood, or to trauma only. The motor functions were evaluated using the BBB score for open-field ambulation (**A**), the KSAT score for swimming (**B**), a grid walk test (**C**), and a beam walk test (**D**). Pain thresholds were assessed by analyzing the avoidance to thermal (**E**) and mechanical (**F**) stimuli to the sole of the left and right hind paws. Thermal thresholds are expressed as the delay to the avoidance response while mechanical thresholds measured the pressure when avoidance occurs. Data are presented as the mean ± SD, *n* = 6–8. In the time line at the top of the figure, MT indicates the complete set of motor tests, while ST indicates sensory tests

To determine if the expanding cysts were associated with functional deficits in addition to what is caused by the traumatic insult per se, we compared motor and sensory functions in PTS rats, i.e., mild contusive trauma combined with blood (trauma + blood) to the control group with only contusive trauma (trauma). One week after injury, the average BBB score dropped from 21 (normal) to 13–14. The score improved to an average of 16 the following week and remained there for the entire 20-week period. There was no sign of decline in hindlimb function during the latter part of the test period when cyst expansion takes place (Fig. 6A). Rats that only received the contusion injury showed an almost identical change in BBB score over time. Two-way repeated measures ANOVA revealed no significant main effect of time and injury type on behavioral score and no significant interaction between time and injury type (Table 1). A similar response was seen for swim performance. The KSAT score dropped from 19 (normal) to 12–13 the week after injury, recovered to 16–17 at week two, and remained at this level (Fig. 6B) with no statistical difference between rats who had undergone trauma and subarachnoid blood injection and those who had undergone trauma alone (Table 1). The two other motor tests, assessing foot slips during grid walk and scoring of beam walk, showed similar results (Fig. 6C, D) (Table 1). Functional deficits in hindlimbs are primarily due to loss of white matter, while the cysts in our PTS model mainly expand in the gray matter. This is also reflected by the complete lack of correlation between BBB and KSAT scores and cyst volumes (Suppl. Fig. 4).

**Table 1:** Results of statistical analysis of the functional tests analyzing motor and sensory functions in rats receiving traumatic injuries to the thoracic spinal cord, with or without subarachnoid injections of blood to elicit PTS

Motor tests			
BBB		Grid walk	
Time	F(4,20) = 3,18, *p* > 0.05	Time	F(4,20) = 1.51, *p* > 0.05
Injury	F(1,5) = 1.75, *p* > 0.05	Injury	F(1,5) = 0.415, *p* > 0.05
Time × injury	F(4,20) = 0.493, *p* > 0.05	Time × injury	F(4,20) = 1.15, *p* > 0.05
KSAT		Beam walk	
Time	F(4,20) = 1.46, *p* > 0.05	Time	F(4,20) = 2.05, *p* > 0.05
Injury	F(1,5) = 1.27, *p* > 0.05	Injury	F(1,5) = 1.58, *p* > 0.05
Time × injury	F(4,20) = 1.15, *p* > 0.05	Time × injury	F(4,20) = 0.092, *p* > 0.05
Sensory tests			
Thermal threshold–left		Thermal threshold–right	
Time	F(3,15) = 0.469, *p* > 0.05	Time	F(3,15) = 0.300, *p* > 0.05
Injury	F(1,5) = 1.14, *p* > 0.05	Injury	F(1,5) = 2.20, *p* > 0.05
Time × injury	F(3,15) = 1.22, *p* > 0.05	Time × injury	F(3,15) = 1.68, *p* > 0.05
Pressure threshold–left		Pressure threshold–right	
Time	F(3,15) = 2.68, *p* > 0.05	Time	F(3,15) = 6.81, *p* < 0.05
Injury	F(1,5) = 0.137, *p* > 0.05	Injury	F(1,5) = 0.137, *p* > 0.05
Time × injury	F(3,15) = 1.22, *p* > 0.05	Time × injury	F(3,15) = 1.68, *p* > 0.05

The change in pressure threshold in the right hind paw over time was the only statistically significant difference

For sensory testing, we only observe a unilateral change over time in the right paw for avoidance to mechanical stimuli, but with no significant differences between injury types in avoidance response to either mechanical or thermal stimuli (Table 1; Fig. 6E, F).

### Cell Transplantation to the Rat PTS Model

We next investigated if it was possible to inject small neurospheres (hNPCs) and dissociated cells (hNESCs) into the PTS cysts and achieve long-time survival of the transplanted human cells. Rats with identifiable cysts on MRI received 300,000 cells into T2*-hyperintense cysts, 10 weeks after injury. During surgery, the cysts could sometimes be seen as dark shadows below a thin layer of the remaining tissue. When the wall of a cyst was punctured to inject cells, a pulsating flow of cyst fluid was often seen. Through the use of glass capillaries for cell injection, we could monitor the transfer of neurospheres (hNPCs) or cell suspensions (hNESCs) into the cyst. It was however not possible to close the slit in the cyst wall, and we could consequently not eliminate the risk of leakage of transplanted cells after the wound was closed.

**Fig. 7:**
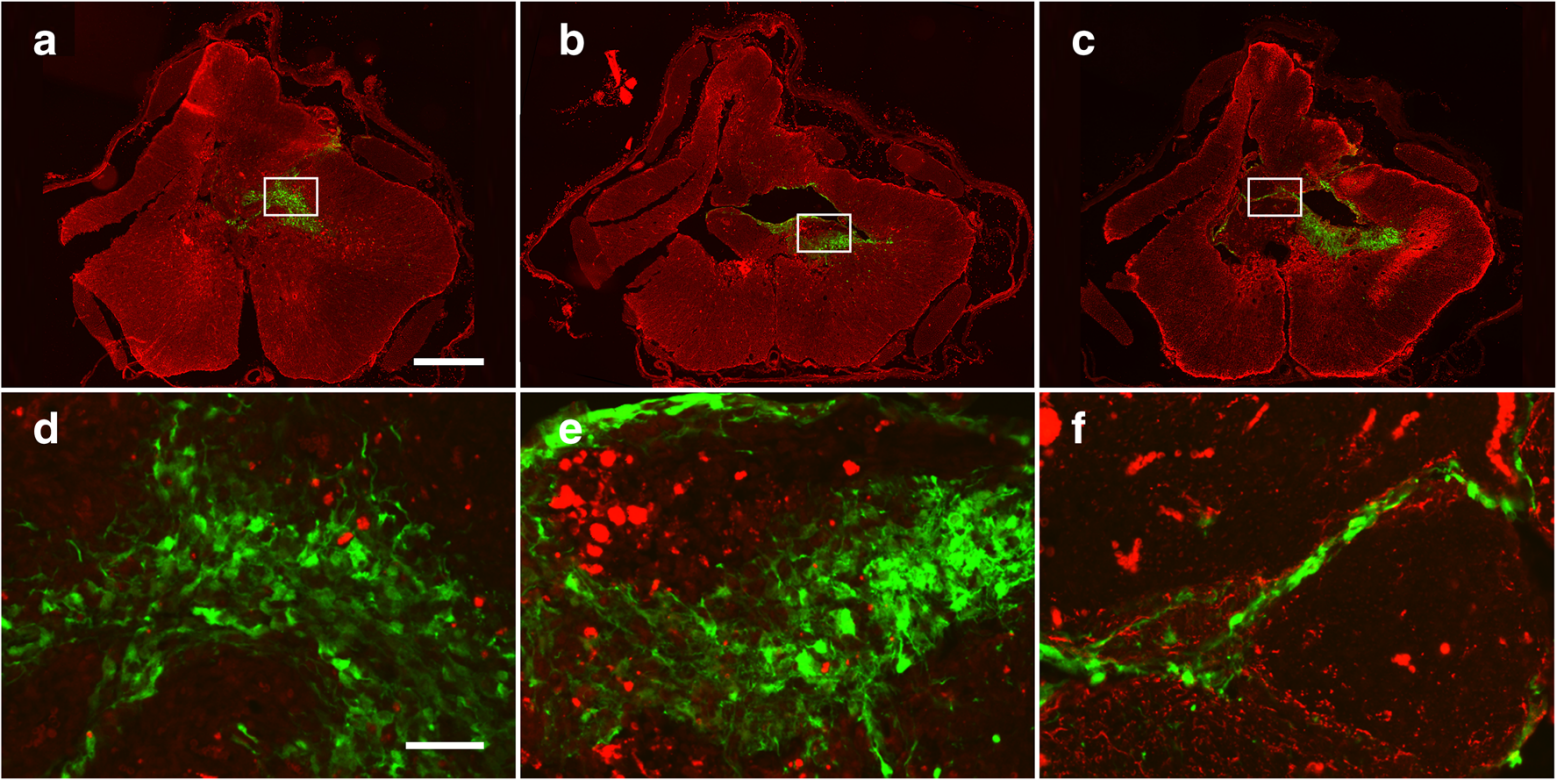
Sections of a spinal cord showing a cyst 20 weeks after injury with GFP-expressing hNPCs 10-week post-transplantation, immunostained for GFAP (red) to identify astrocytes in the host tissue and the graft. The boxed areas in the low magnification images (**A**–**C**) are shown at higher magnification (**D**–**E**). Grafted hNPCs were found in large numbers at the rostral end of the cyst (**A**, **D**). They covered most of the cyst walls, and had also migrated into the surrounding host parenchyma, forming a large cluster of grafted hNPCss (**B**, **E**). Parts of the cyst had disappeared, and a thin membrane of grafted cells were found at the position of these parts, probably representing merged, previously opposing cyst walls lined with hNPCs. Scale bars represent 200 μm (**A**) and 25 μm (**D**)

In rats with large spinal cord cysts, transplantation was usually associated with partial collapse of the cyst. The cysts in rats with engrafted hNPCs or hNESCs typically displayed a thin layer of human cells covering most of the cyst walls. Aggregates of human cells were often located at the caudal and rostral ends of cysts filling these parts of the cyst with neural tissue (Fig. 7A, D), while in the central parts, cells covered much of the cyst walls with a thin cell layer, with cells also in the parenchyma surrounding the cysts (Fig. 7B, E). We found numerous examples where the opposing cyst walls had merged, with thin sheets of human cells in between (Fig. 7C, F), but occasionally cyst walls had merged with no remaining human cells between.

**Fig. 8:**
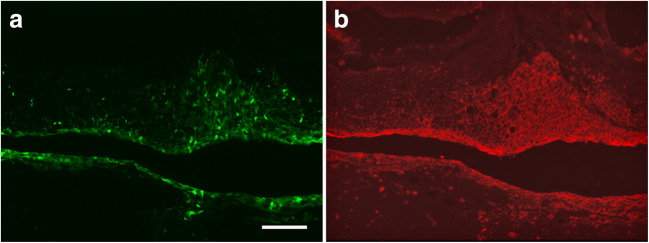
Section through a PTS spinal cord with transplanted GFP-expressing hNESCs, stained for the stem cell marker Sox2. Arrows indicate a group of human GFP-expressing cells adjacent to the remaining cyst cavity that are Sox2-immunoreactive. Arrowheads show dispersed GFP-expressing cells that have migrated into the surrounding parenchyma which are also Sox2-positive. In addition, numerous rat neurons in the vicinity of the cyst are Sox2-positive

In all rats with surviving transplants, human cells had migrated into the dorsal and/or ventral gray matter. The grafted human cells were immunoreactive to the neural stem cell markers nestin and (Fig. 8) sox-2 (Suppl. Fig. 5), while β-tubulin III and GFAP, markers for neurons and reactive astrocytes, respectively, were absent (Fig. 7D–F). This shows that differentiation of the engrafted hNPCs and hNESCs had not yet taken place 10–12 weeks after transplantation.

An important finding was that the smallest cystic changes (< 2 mm^3^) at 10 weeks after injury had disappeared at 20 weeks, irrespective of whether they received a transplant or not. Hence, this volume was used in the evaluation of all experiments as a criterion to define what we consider a cyst, excluding small degenerative cysts that often appear at the lesion site of SCI in rats [27].

### Effects of Cell Therapy on Cyst Volume

**Fig. 9:**
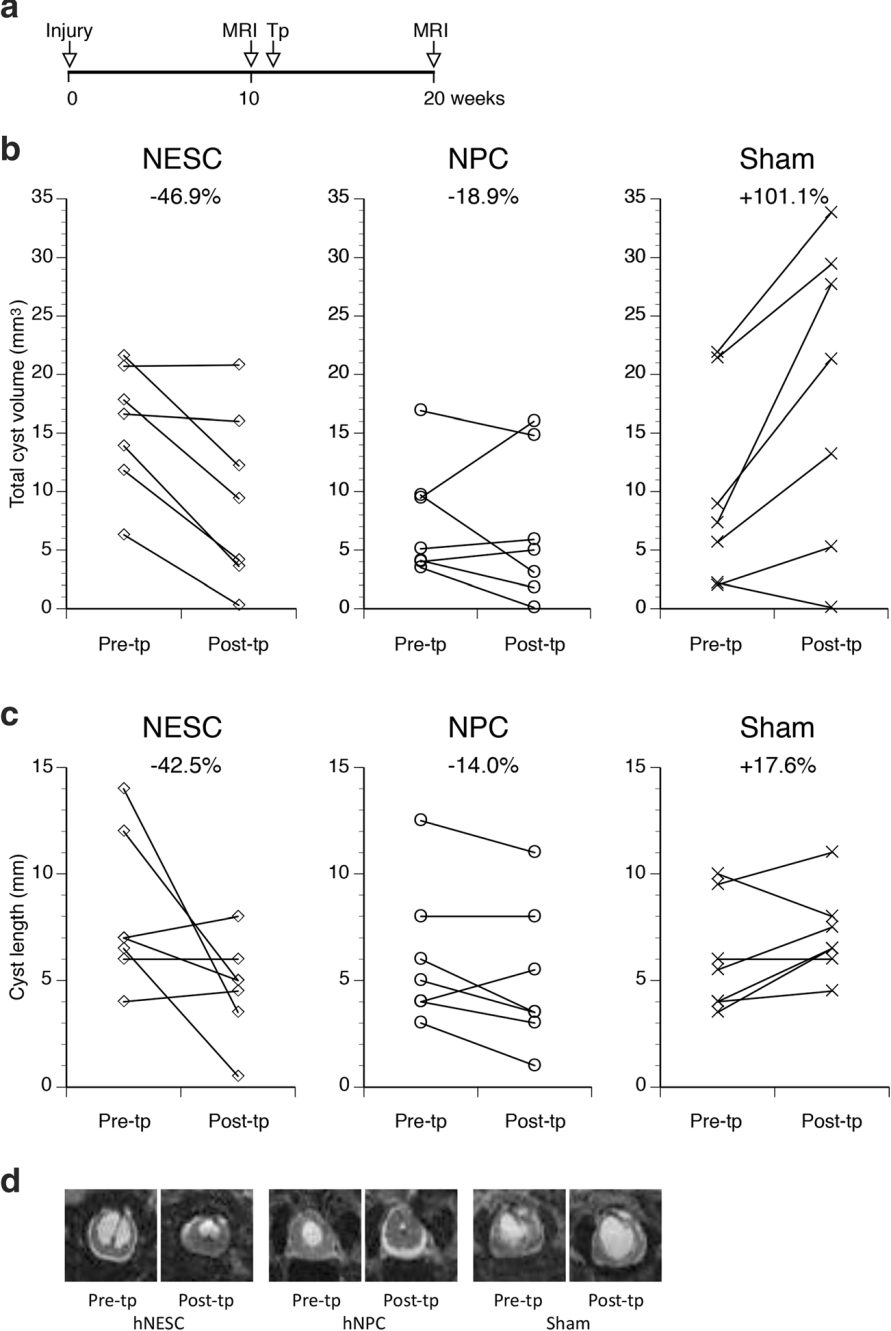
Measurements of sizes of T2*-weighted hyperintensive cysts in PTS rats before and after transplantation with hNESCs, hNPCs, or subjected to sham surgery by injection of the medium used for the cell transplantation. (**A**) The time line shows when the injuries, MRI and cell transplantation (Tp) were performed. (**B**) Volumes and (**C**) cyst lengths were measured 10 weeks after injury, just prior to transplantation or sham transplantation (Pre-tp), and again 10 weeks after transplantation (Post-tp) or sham, and the results for each rat plotted. Both cyst volumes and lengths were significantly lower in the groups subjected to cell transplantation, compared to sham-transplantation (*p* < 0.01, ANOVA for repeated measures, post-hoc Bonferroni test). D) MRI images of representative examples of rats from the three experimental groups showing cysts before and after transplantation or sham

In rats subjected to the combination of mild trauma and injection of 100 μl of blood, MRI 10 weeks after injury showed cysts ≥ 2 mm^3^ in 75% of the rats. After subsequent transplantation followed by another 10 weeks before the next MRI, the sham-transplanted rats on average exhibited almost exactly a doubling of the cyst volume (+ 102%), whereas the volumes in hNPCs-transplanted rats decreased on average by − 18.8%. The effect of hNESC transplantation on cyst volumes is even larger with a reduction of cyst volumes by − 46.8% (Fig. 9, upper panels). Both cell types showed highly significant effects on cyst volumes (*p* = 0.00014, *f* = 14.7, df = 2,19), with no significant difference between rats treated with hNESCs and hNPCs. When we analyzed the rostrocaudal expansion of the cysts, the length of cysts in the sham group increased by + 17.6% between 10 and 20 weeks post-injury, and both cell types completely prevented the expansion, reducing the cyst lengths by − 14.0% (hNPCs) and − 42.5%, (hNESCs) (Fig. 9, lower panels), a significant reduction of the cyst lengths (*p* = 0.0097, *f* = 5.89, df = 2,20).

While the effects on cyst volumes were rather consistent within the two treatment groups, we found a large variation in the number of surviving cells 10 weeks after transplantation, with low number of surviving cells in several rats. The median number of human cells were 5669 (interquartile range 504–94,160). In 2/14 graft recipients, we found no surviving cells at all. Still, also in these animals, there were major reductions of cyst volumes, showing that the reduction of cyst volumes was not due to the grafted cells filling up the cysts. To further show that the transplanted cells prevent cyst expansion rather than filling the cavity with cells, we calculated the total volume of engrafted cells based on the number of surviving human cells and measurements of average cell volume determined from serial confocal optical sectioning of GFP-expressing cells. With the calculated mean cell volume of 490 μm^3^, the lower and upper quartile of human cells correspond to 0.0002 and 0.046 mm^3^, respectively. Even 1 million surviving human cells would only occupy a volume of ≈ 0.5 mm^3^, an order of magnitude less than the cyst volume reductions in the transplanted rats. Thus, our data show that transplanted hNPCs and hNESCs prevent further expansion of the cysts in rats with PTS.

## Discussion

The aim of this study was to use an animal model of PTS that closely mimics the clinical situation and investigate if cell therapy is a potential treatment for this condition. We applied mild traumatic injuries to the lower thoracic spinal cord of rats with subarachnoid injections of autologous blood and found that the majority of rats displayed extracanalicular cysts that were similar to PTS in SCI patients, while chronic cysts only appeared in ≈ 20% of rats exposed to the mild injury alone. As the first proof-of principle of neural cell therapy in PTS, we also show that the chronic expansion of these cysts could be completely prevented by transplantation of either of two types of human neural stem/progenitor cells, fetal neural precursor cells (hNPCs), and neuroepithelial-like stem cells (hNESCs) derived from induced pluripotent stem cells (iPSC).

In previous animal models, various features of PTS have been artificially recreated to demonstrate possible pathophysiological mechanisms. Although PTS occasionally can be seen in rats after experimental SCI [18, 28], more reliable models of PTS are necessary. Cho et al. [14] recognized that subarachnoid scarring and tethering after SCI probably affect CSF flow and used injections of the mineral kaolin into the subarachnoid space of rabbits in conjunction with a traumatic injury to achieve a similar reaction. The injections of kaolin triggered local inflammation—arachnoiditis—and tethering, and the rabbits consistently developed non-communicating (extra-canalicular) spinal cord cysts similar to the clinical condition. Rat models using intraparenchymal kaolin injections [29]or subarachnoid injections combined with clip compression injury [30] were later developed to study pathophysiological mechanisms and aspects of PTS. Together with clinical data, these experimental results pointed at arachnoiditis being a key component in the pathophysiology of PTS [31, 32]. Other animal models were based on spinal lesions using the excitotoxic glutamate receptor agonist quisqualic acid, in combination with subarachnoid injection of kaolin [15, 16]. Although excitotoxicity is an important process during the acute phase of traumatic SCI [33], a number of processes such as hemorrhage, shearing of tissue, and direct injury of axons do not occur in the excitotoxic model.

However, when we used kaolin to induce PTS in rats, post-mortem we found kaolin in several cysts, remaining more than 10 weeks after injection. If cells are injected into such cysts, they will most certainly be affected by kaolin, making interpretation of the experiments very difficult. Since kaolin is used to trigger the arachnoiditis, as an alternative, we decided to deposit autologous blood in the subarachnoid space to induce arachnoiditis and tethering. Free blood induces inflammation in the CNS, and the association to PTS has been clinically demonstrated by reports of syringomyelia appearing after subarachnoid hemorrhage, with extensive arachnoiditis and tethering without associated trauma [34, 35]. The subarachnoid injection of blood was combined with a mild spinal contusion of the lower thoracic cord. Among all experimental animals, 50–75% of the rats had developed chronically expanding cysts 10–12 weeks after injury, the higher incidence in the rats given 100 μl of blood in the intervention study. In addition to these rats with expanding cysts, some rats had fluid-filled cystic changes smaller than 2 mm^3^ 10 weeks after injury. During the following 10 weeks, they disappeared spontaneously; we therefore decided to only include cysts ≥ 2 mm^3^ in our definition of PTS cysts.

Post-mortem we found several examples of extensive tethering of the spinal cord to the surrounding dura mater. Microscopy showed that the cysts were usually located rostral to the lesion, often with several lobes. In most cases, the cysts extended over two segments or more, a common definition of syringomyelia in patients [3, 36] and animal models [30]. The cysts were lined with reactive astrocytes labeled by GFAP, similar to human PTS [37], and did not represent a widening of the central canal (lined by ependymal cells). Thus, these cysts—produced by mimicking pathophysiological features of PTS—were in many aspects similar to the cysts found in PTS patients and should serve as a useful animal model of PTS.

The clinical diagnosis of PTS depends on MRI to analyze the size, location, and expansion of the cyst, so it was important that our PTS model could also be analyzed with MRI. When we compared the T2*-weighted MRI images to tissue sections, hyperintense regions corresponded as expected to empty cysts, and tissue septa in branched cysts could clearly be identified, similar to a previous study [38]. However, we also found T2*-hypointense regions adjacent to hyperintense cyst, which corresponded to areas with tissue debris and ED1-positive macrophages/microglia. The presence of iron/hemosiderin in regions with hypointense MRI signals was confirmed in Fe-stained sections. We found long, narrow T2*-hypointense structures rostral and sometimes caudal to T2*-hyperintense cysts that corresponded to hemosiderin surrounding the central canal. Noble and Wrathall [39] reported that hemorrhage may occur around the central canal several millimeters from experimental SCI lesions. The hemosiderin we found more than 10 mm rostral to a small contusion suggests that subependymal hemorrhage or extravasation may be part of the cyst expansion. In contrast, clinical PTS cysts are empty on pathological examination, and hemosiderin-containing macrophages and microglia are instead found in the walls of the cysts [39]. The presence of debris in our experimental PTS cysts may be due to the relatively short time after injury (≈ 20 weeks), while clinical PTS often is diagnosed many years after SCI [3, 36].

In the PTS model, we used mild injuries from which the rats would be expected to recover within 3–4 weeks, increasing the chance of detecting functional deterioration as the cysts developed during the chronic phase. However, in none of the tests did we find functional consequences of the developing cysts per se. With regard to motor functions, this is not surprising considering that the cysts were located in the thoracic gray matter. Hindlimb motor functions in rodents correlate closely to the loss of thoracic white matter, but not gray matter [20, 21] (see [40]for discussion). Our data imply that the radial expansion of the cysts does not involve the white matter to any major extent, in agreement with the structural data from MRI and microscopy. In addition, we found no changes in mechanical or thermal pain thresholds, while Seki and Fehlings [30] reported lowered pain thresholds 6 weeks after trauma with subarachnoid kaolin injection. However, it is not clear which dermatomes they analyzed. It is possible that kaolin induced inflammation in the dorsal root ganglia, which may have contributed to the change in pain thresholds through increased excitability of sensory ganglion neurons [41]. More refined analyses of motor and sensory functions are necessary to determine if our rat PTS model also can be used to analyze functional effects of expanding spinal cord cysts.

We then used the rat PTS model to study the potential of cell therapy to prevent cyst expansion. There are numerous studies in SCI on transplanted human stem and progenitor cells isolated from fetal CNS. hNPCs improve functional outcome after experimental SCI, mainly through a neuroprotective effect on host neurons [17]. These cells have an advantageous safety profile, with no signs of tumor formation [17, 42]. In a series of studies, the Tuszynski group showed that human fetal spinal cord-derived stem/progenitor cells as well as iPSC-derived neural stem cells transplanted to SCI integrate with the host neuronal circuitry and contribute to functional improvement in rodents and primates [43–45]. Recently the first report on a clinical phase 1 trial with similar fetal stem/progenitor cells was published [46]. These studies demonstrate the potential in acute and chronic SCI of the two cell types we used for PTS.

In the first transplantation experiments, we showed survival and tissue approximation of stem/progenitor cells injected into the cysts guided by pre-operative MRI. Microscopic analysis revealed that transplanted human cells were organized in the spinal cord in four ways: (1) Small numbers of cells—sometimes just a single layer—were found between what apparently were opposing walls of a collapsed cyst, (2) small aggregates of cells in the partially collapsed cysts, (3) cells that had migrated into the surrounding host parenchyma, and (4) in a few rats (< 10%) we found one large cell aggregate, filling up parts of the cyst. Usually cell patterns 1–3 were seen in the same rat. Importantly, we never found signs of tumor growth, i.e., that the human tissue compressed and/or displaced the host tissue.

We then performed an intervention study to determine if neural cell therapy can be used to reverse the expansion of intraspinal cysts, the therapeutic effect that is the prime interest for the PTS patient. In our rat PTS model, we found that the expansion of the cysts was not only prevented in transplanted rats, but in fact reversed. hNESCs and hNPCs reduced the average cyst volume by close to − 50% and − 20%, respectively, while the cysts in sham-transplanted rats doubled in size over the same 10-week period. The reduced cyst sizes in transplanted rats displayed as reduced lengths of the cysts by − 43% and − 14%, while cysts in the sham group increased by 18% in length. To our knowledge this is the first proof-of-concept in an animal model that human neural cell therapy can be used to prevent the progressive cyst expansion in an animal model of PTS.

The large effect on cyst volumes was even more surprising in light of the low number of human cells present 10 weeks after transplantation. There was no correlation between the number of human cells present and cyst volume reductions. The graft recipients with no remaining human cells after 10 weeks suggest that the therapeutic effect of the human cells was exerted during the first weeks after transplantation. Our cell volume measurements also showed that only a minute part of the cyst volume reductions was achieved by replacing cyst fluid with human cells. To reduce the cyst volumes significantly by filling them with cells would have required orders of magnitude more cells than were transplanted in our study. The microscopic analysis suggested that the transplanted cells adhere to the walls of the collapsed cyst, possibly already within hours after transplantation. The cysts collapse when the small surgical slit is made, but while sham-treated cysts regain their shape and continue to expand, in cysts with transplanted cells, this is largely prevented. Our results suggest that even a thin layer of human cells could adhere to the opposing walls of the cyst to merge them, thereby preventing re-filling of the cyst and further expansion. In the few rats with no surviving human cells after 10 weeks, the grafted cells could still bring about merging of the cyst walls during the first days-weeks, which could be retained by adhesion between the astrocytes in the cyst walls. This fits with the consistent prevention of cyst expansion in spite of the relatively small number of human cells remaining 10 weeks after transplantation. Other explanations are of course also possible, as alternatives or in combination with cell adhesion.

The reason for the low number of surviving human cells 10 weeks after transplantation is not clear. It has been shown previously that large numbers of transplanted cells, in particular dissociated cells, die acutely after transplantation [47, 48], even with adequate immune suppression. Cells could have been flushed out of the cyst before they attached to the interior of the cyst. It is also possible that the interior of the cysts is a hostile environment for stem/progenitor cells. Long-term survival of transplanted human fetal tissue has been shown in patients with PTS [10, 11], but the situation may be different for stem/progenitor cells.

Surprisingly, we found that neither hNPCs nor hNESCs, in the cyst or in the surrounding parenchyma, had differentiated 10 weeks after transplantation. Although even longer time was required for differentiation of neural progenitor cells derived from human embryonic stem cells [49], our results differ from previous studies on the same [17, 50], and similar [43] cells transplanted to subacute or chronic SCI. We can only speculate as to what mechanisms are involved. However, it was obviously not necessary that the transplanted cells differentiated to reduce the cyst volume. Still, many more remaining, differentiating transplanted cells will be required to achieve our secondary goal to replace a significant part of the lost tissue and neuronal circuits, in addition to the successful prevention of cyst expansion.

We did not perform detethering of the injured spinal cord in conjunction with the transplantation surgery. From a clinical perspective, it is interesting that the strong treatment effects we observed occurred although abundant tethering was still present. This suggests that transplantation of neural stem/progenitor cells may have a significant effect also in clinical situations with extensive tethering that cannot be cleared, i.e. in patients with the highest risk of recurrence [8]. The effects of hNESCs and hNPCs were not significantly different, but it is noteworthy that iPS cell-derived neural stem cells had a larger effect than immortalized human fetal stem/progenitor cells in a recent study on SCI [51].

To conclude, we established a novel rat model of PTS with a progressive cyst expansion over several months without the use of foreign material or drugs, closely mimicking the human condition. We used this model to show that two types of human neural stem/progenitor cells, hNPCs and hNESCs, can be transplanted into the PTS cysts, with long-term survival of these human cells, although showing limited differentiation. We provide proof-of-concept for these human neural cells as effective treatment of PTS, and it is possible that after proper safety studies, either of these cell types could be a candidate for a clinical trial.

## Supplementary Information

Figure S1 Tissue sections from three control rats 20 weeks after mild trauma without subarachnoid injections of blood. The upper row show representative htx-eosin stained sections (A-C), and the middle row adjacent sections stained with GFAP to show reactive astrocytosis (D-F). The three rats had according to the MRI analysis total cyst volumes of 0, 0.42 and 1.35 mm^3^. The lower row shows the degenerative changes taking place at 2 (G), 8 (H) and 20 (I) weeks after trauma, as illustrated by sections from representative rats. Scale bars correspond to 400 μm. (PDF 86892 kb)

Figure S2 Box plot of cyst volumes in rats exposed to mild contusive trauma only (Trauma) and trauma combined with subarachnoid injection of 30 μl autologous blood (Trauma + Blood). Due to shrinkage of tissue during fixation, the volumes were normalized to the median volume of the trauma only-group. Data are expressed as median with quartiles, and 10 and 90 percentiles indicated. *p < 0.05, Mann-Whitney U-test. (PDF 13 kb)

Figure S3 Sagittal projections of T2-weighted MRI images of the thoracic spinal cord of 2 rats, 18 weeks after inducing post-traumatic cysts using mild contusive trauma and subarachnoid injection of 30 μl of blood. The arrows in the left picture indicate the location of the contusion, vertebra T9 (spinal segment T11). Light, hyperintense regions representing fluid-filled cysts occupy the segments immediately rostral to the injury, while the right picture shows a long dark hypointense region in the rostral part (arrow). (PNG 425 kb)

High resolution image (TIFF 857 kb)

Figure S4 Cyst volumes plotted against BBB motor score (left graph) and KSAT swim score (right panel) for rats subjected to mild contusive trauma (Trauma) or trauma combined with subarachnoid injection of 30 μl autologous blood (Trauma + Blood). (PDF 22 kb)

Figure S5 Section through a PTS spinal cord with transplanted GFP-expressing hNESCs, double stained for the stem cell marker Sox2. Arrows indicate a group of human GFP-expressing cells adjacent to the remaining cyst cavity that are Sox2-immunoreactive. Arrowheads show some of the dispersed GFP-expressing cells that have migrated into the surrounding parenchyma which are also Sox2-positive. In addition, numerous rat neurons in the vicinity of the cyst are Sox2-positive. (PNG 623 kb)

High resolution image (TIF 903 kb)

ESM 1 (PDF 1533 kb)
