# Supplementary figures and images for: Transplantation of Human Neural Precursor Cells Reverses Syrinx Growth in a Rat Model of Post-Traumatic Syringomyelia
Source: Neurotherapeutics. 2021 Jan 19;18(2):1257–72. doi: 10.1007/s13311-020-00987-3 (PMC8423938; doi:10.1007/s13311-020-00987-3)

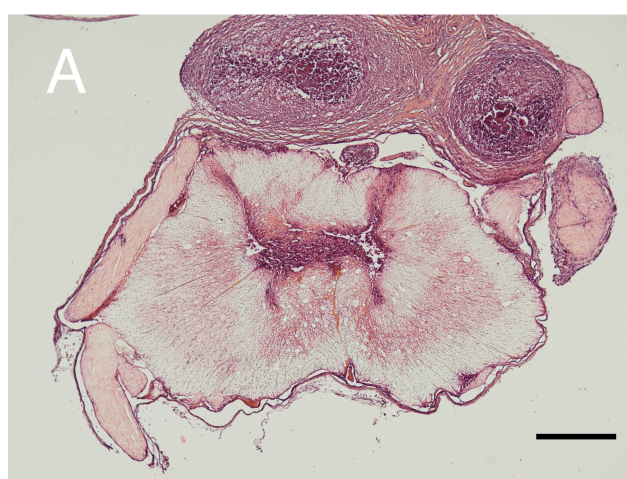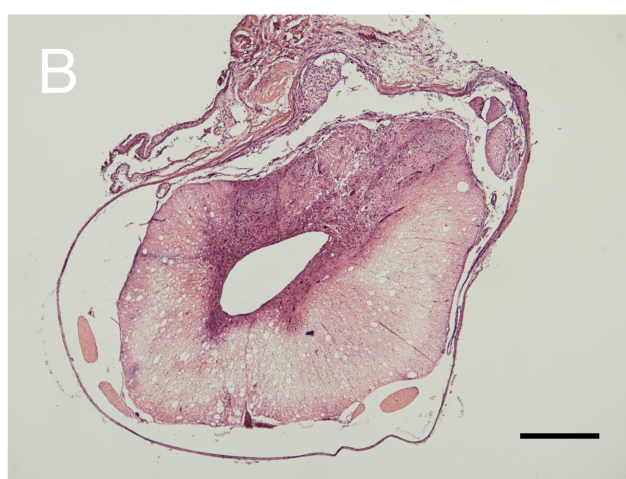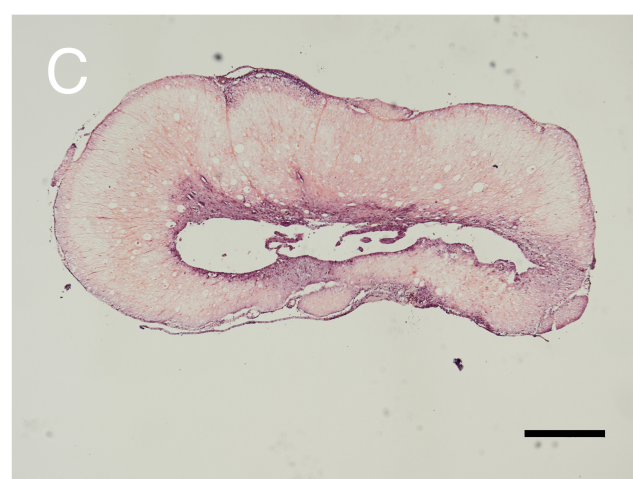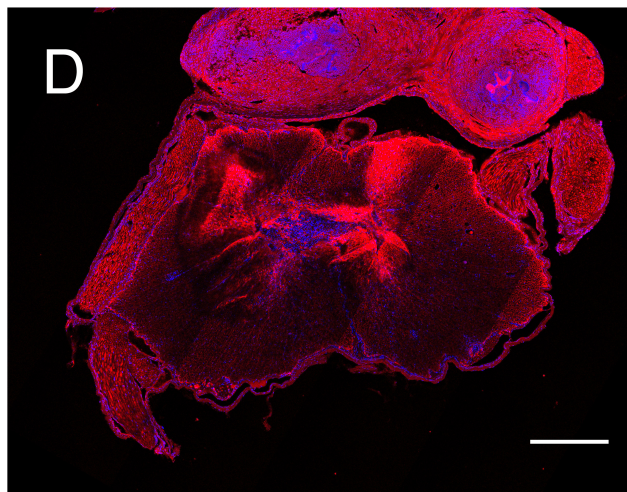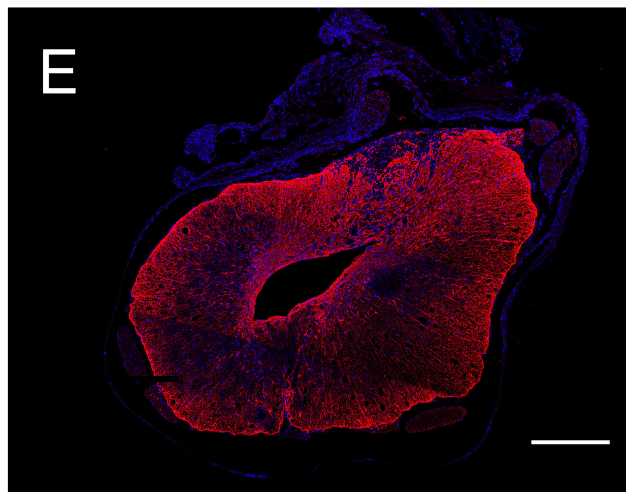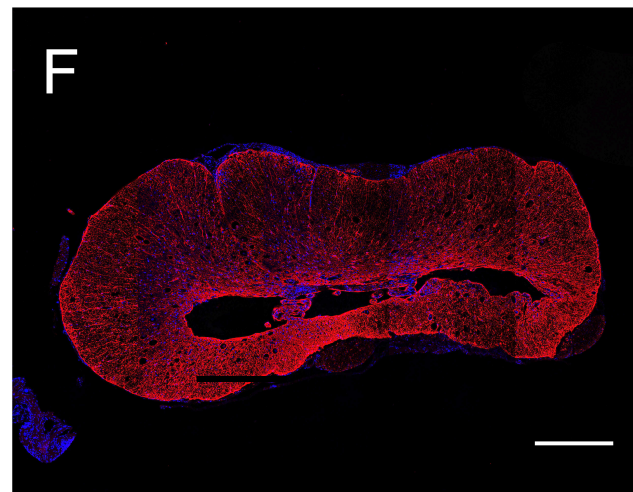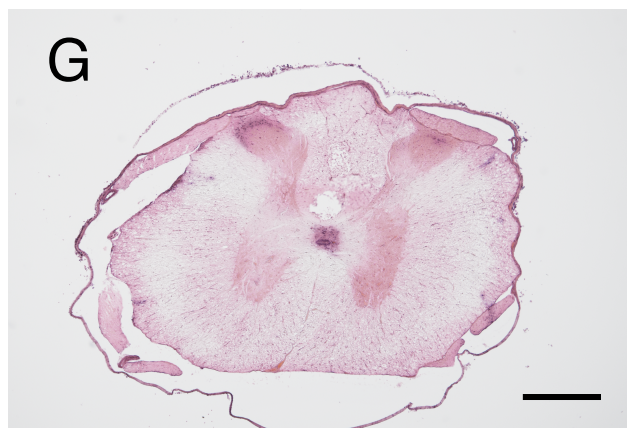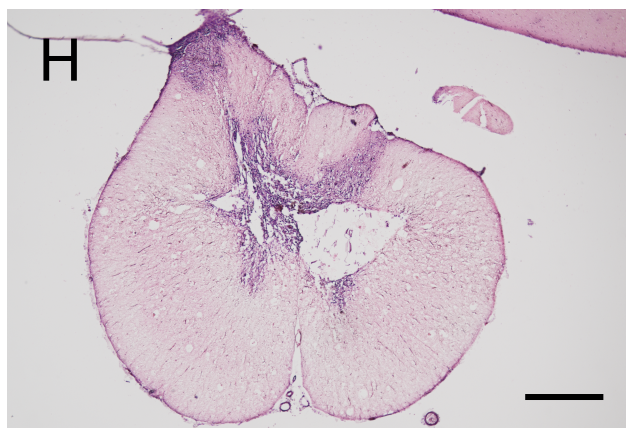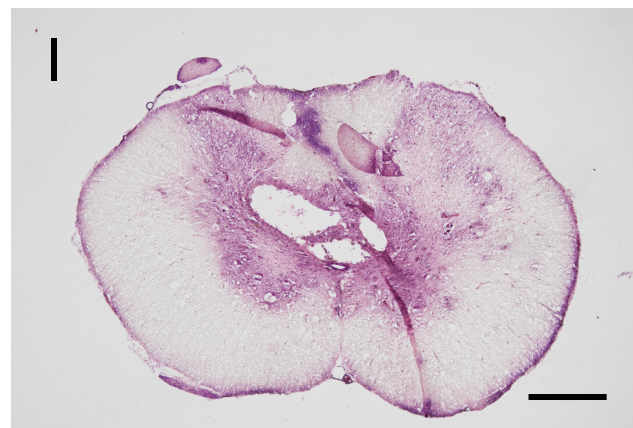

Supplement: Supplementary file 1 — Tissue sections from three control rats 20 weeks after mild trauma without subarachnoid injections of blood. The upper row show representative htx-eosin stained sections (A-C), and the middle row adjacent sections stained with GFAP to show reactive astrocytosis (D-F). The three rats had according to the MRI analysis total cyst volumes of 0, 0.42 and 1.35 mm3. The lower row shows the degenerative changes taking place at 2 (G), 8 (H) and 20 (I) weeks after trauma, as illustrated by sections from representative rats. Scale bars correspond to 400 μm. (PDF 86892 kb) [file 13311_2020_987_MOESM1_ESM.pdf]

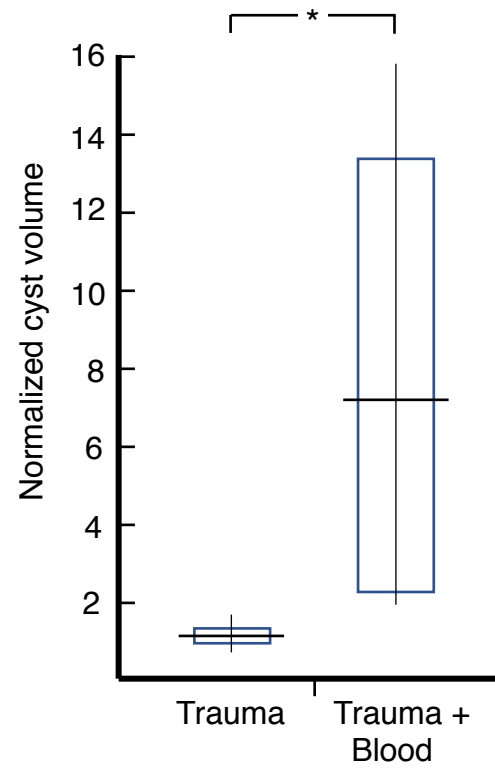

Supplement: Supplementary file 2 — Box plot of cyst volumes in rats exposed to mild contusive trauma only (Trauma) and trauma combined with subarachnoid injection of 30 μl autologous blood (Trauma + Blood). Due to shrinkage of tissue during fixation, the volumes were normalized to the median volume of the trauma only-group. Data are expressed as median with quartiles, and 10 and 90 percentiles indicated. *p < 0.05, Mann-Whitney U-test. (PDF 13 kb) [file 13311_2020_987_MOESM2_ESM.pdf]

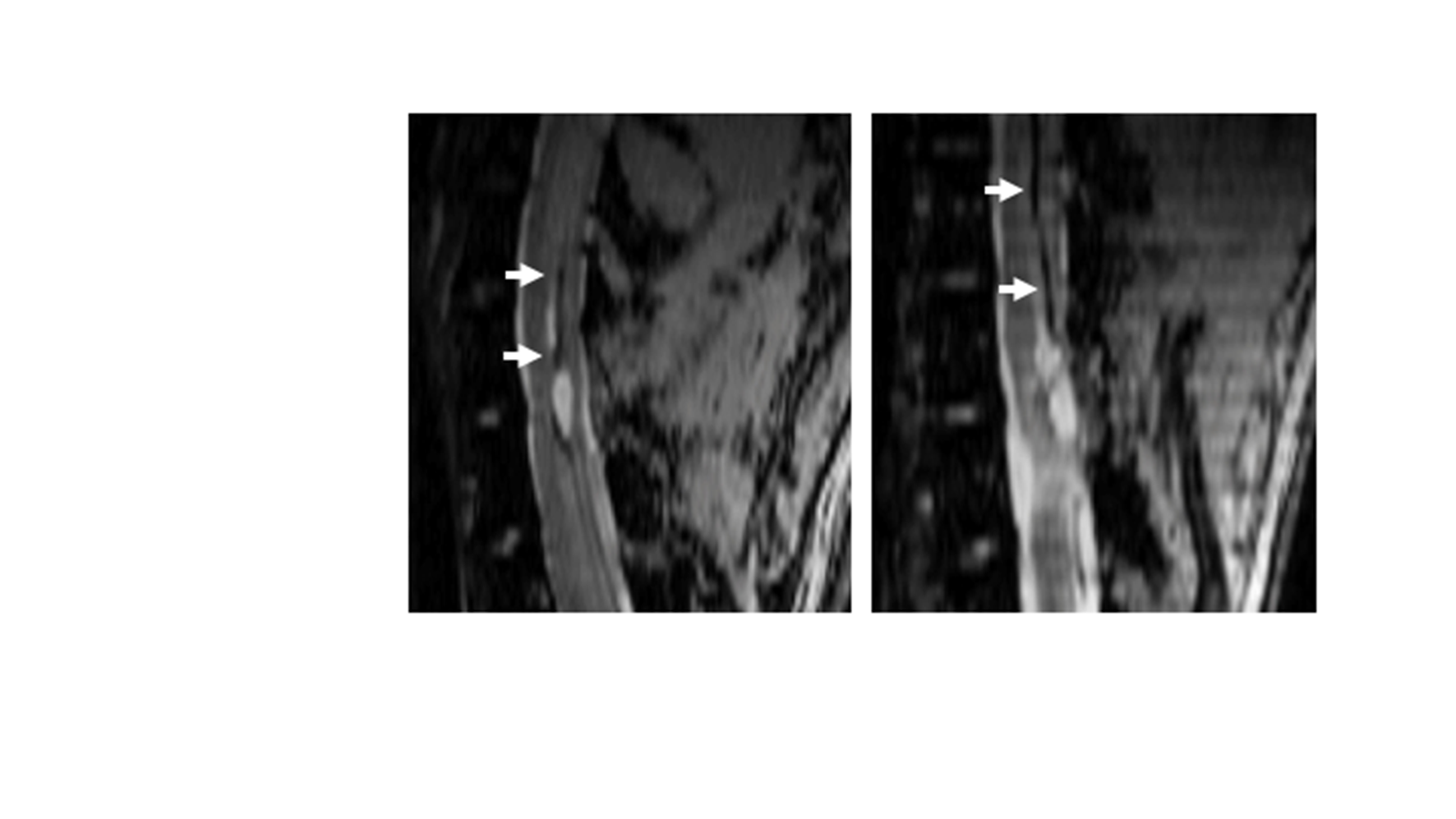

Supplement: Supplementary file 3 — Sagittal projections of T2-weighted MRI images of the thoracic spinal cord of 2 rats, 18 weeks after inducing post-traumatic cysts using mild contusive trauma and subarachnoid injection of 30 μl of blood. The arrows in the left picture indicate the location of the contusion, vertebra T9 (spinal segment T11). Light, hyperintense regions representing fluid-filled cysts occupy the segments immediately rostral to the injury, while the right picture shows a long dark hypointense region in the rostral part (arrow). (PNG 425 kb) [file 13311_2020_987_Fig10_ESM.png]

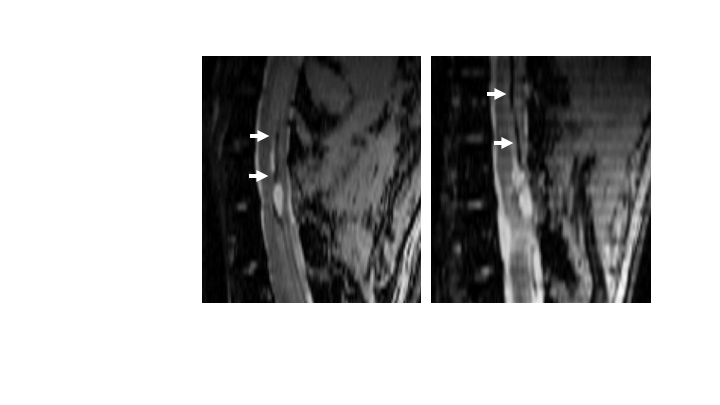

Supplement: Supplementary file 4 — High resolution image (TIFF 857 kb) [file 13311_2020_987_MOESM3_ESM.tiff]

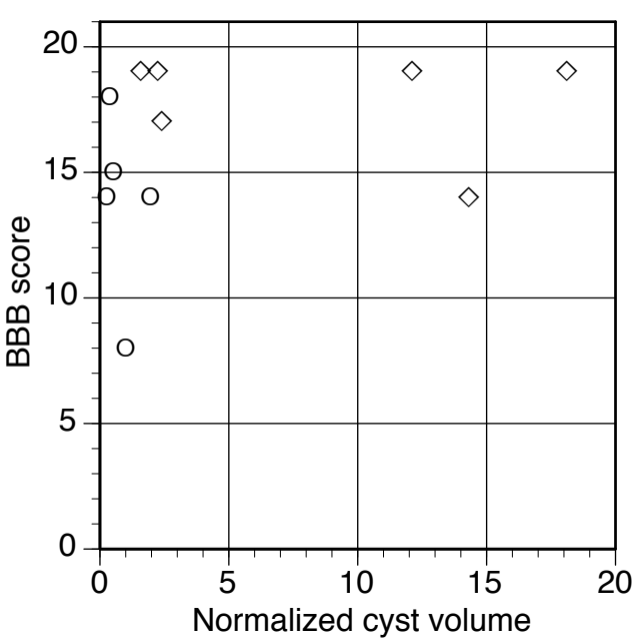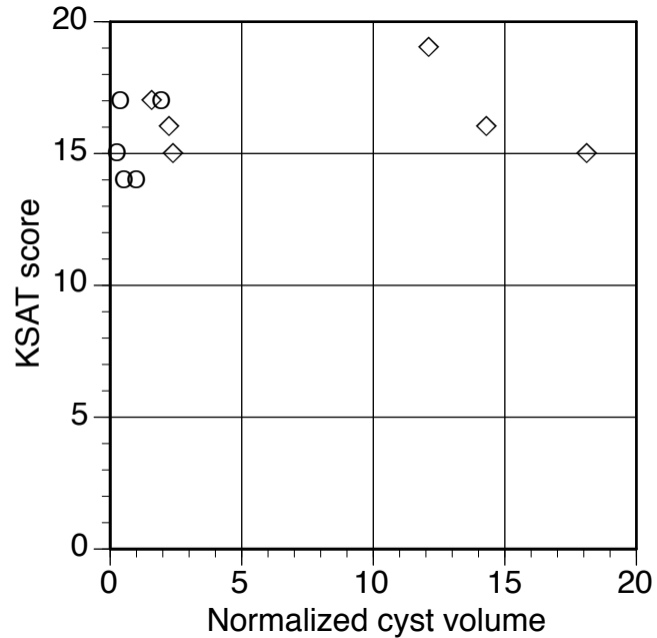

◇ Trauma + Blood  
○ Trauma

Supplement: Supplementary file 5 — Cyst volumes plotted against BBB motor score (left graph) and KSAT swim score (right panel) for rats subjected to mild contusive trauma (Trauma) or trauma combined with subarachnoid injection of 30 μl autologous blood (Trauma + Blood). (PDF 22 kb) [file 13311_2020_987_MOESM4_ESM.pdf]

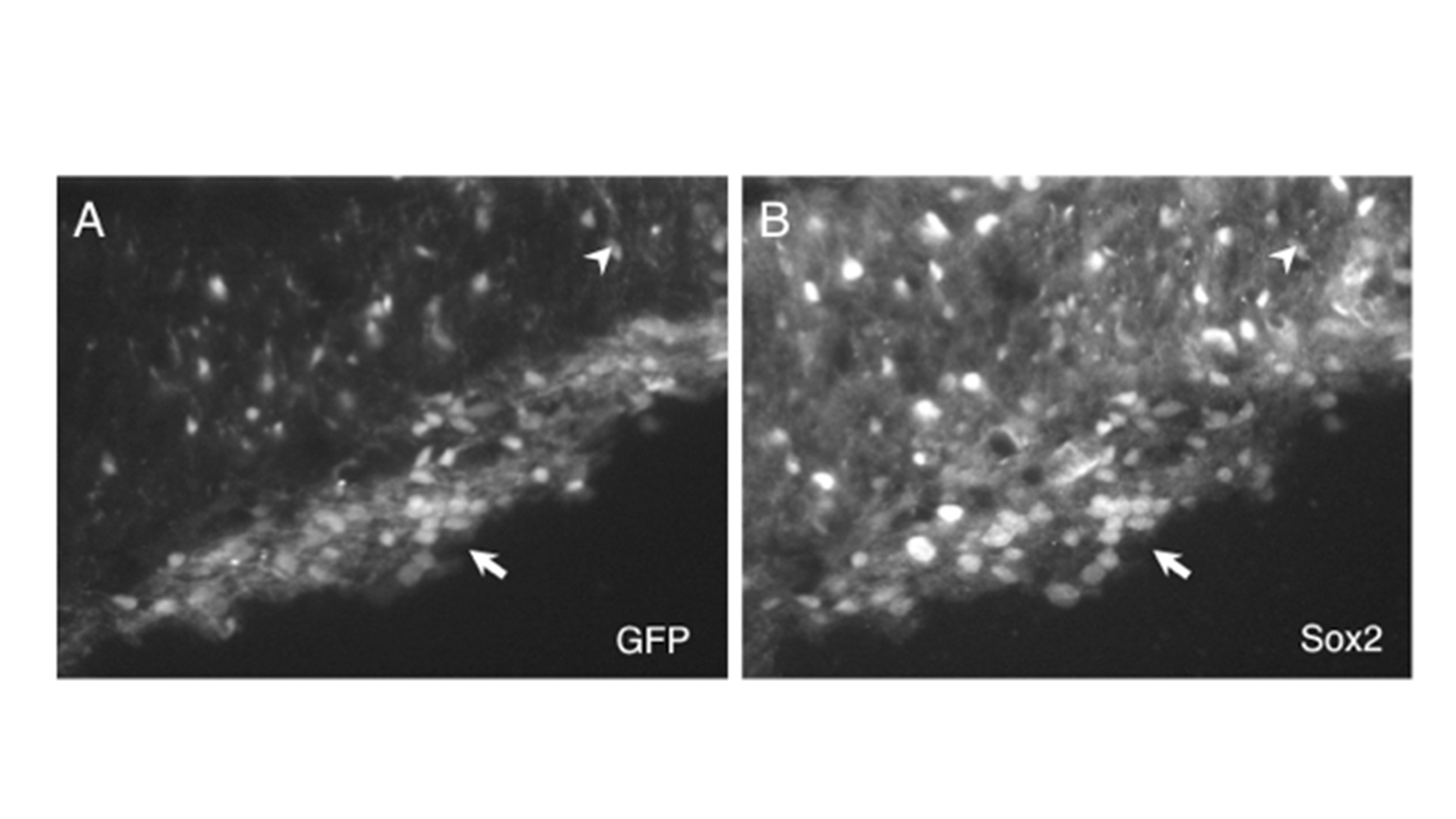

Supplement: Supplementary file 6 — Section through a PTS spinal cord with transplanted GFP-expressing hNESCs, double stained for the stem cell marker Sox2. Arrows indicate a group of human GFP-expressing cells adjacent to the remaining cyst cavity that are Sox2-immunoreactive. Arrowheads show some of the dispersed GFP-expressing cells that have migrated into the surrounding parenchyma which are also Sox2-positive. In addition, numerous rat neurons in the vicinity of the cyst are Sox2-positive. (PNG 623 kb) [file 13311_2020_987_Fig11_ESM.png]

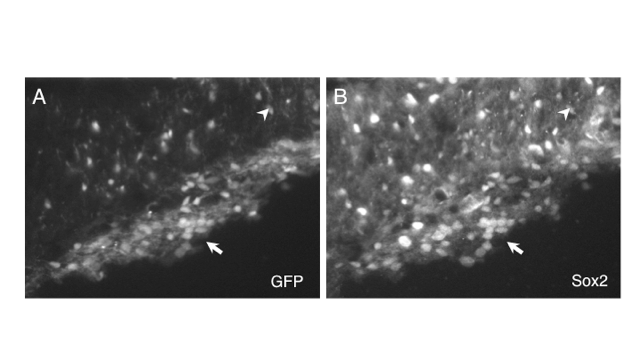

Supplement: Supplementary file 7 — High resolution image (TIF 903 kb) [file 13311_2020_987_MOESM5_ESM.tif]
